# Comparative profiling of amino acids in plasma and serum across children and adults using LC–MS/MS and its implications for precision nutrition

**DOI:** 10.3389/fnut.2026.1850080

**Published:** 2026-07-24

**Authors:** Yanhui Ma, Yi Liu, Yuchan Huangfu, Yunlan Zhou, Yingxia Zheng

**Affiliations:** 1 Department of Laboratory Medicine, Xinhua Hospital, Shanghai Jiao Tong University School of Medicine, Shanghai, China; 2 Faculty of Medical Laboratory Science, College of Health Science and Technology, Shanghai Jiao Tong University School of Medicine, Shanghai, China; 3 Institute of Artificial Intelligence Medicine, Shanghai Academy of Experimental Medicine, Shanghai, China

**Keywords:** age, amino acids, fasting serum, LC–MS/MS, plasma

## Abstract

**Objective:**

Circulating amino acids (AAs) are important biomarkers of nutritional status, metabolic homeostasis, and disease related physiological changes. However, the influence of specimen type and age on AA measurements remains incompletely understood. This study aimed to establish a robust liquid chromatography–tandem mass spectrometry (LC–MS/MS) method for quantifying 29 amino acids (AAs), compare serum and plasma matrices, and investigate age related variations in healthy populations.

**Methods:**

In a cross-sectional study, a total of 231 healthy adults (≥18 years) and 140 children (<18 years) were recruited from the Shanghai area with a subgroup of 26 adults providing paired serum and plasma samples. Twenty-nine AA quantification was performed using LC–MS/MS with 6-aminoquinolyl-N-hydroxysuccinimidyl carbamate (AQC) derivatization. Method validation followed Clinical and Laboratory Standards Institute (CLSI) guidelines for linearity, lower limit of quantitation (LLOQ), precision, accuracy, carryover, and stability.

**Results:**

The assay demonstrated excellent linearity (*R*^2^ > 0.99), precision (CV < 15%), and accuracy (bias < 10%) with minimal carryover and acceptable LLOQs for all 29 AAs. Matrix differences and correlations were evaluated using the paired samples, while age related AA profiles were assessed in largely independent serum and plasma cohorts of pediatric and adult. Serum and plasma concentrations correlated well for most AA. However, significant matrix dependent differences were observed for several amino acids, including GABA, Ser, Tau, Sar, and Asp (*R*^2^ < 0.5). Age related analysis revealed that most essential AAs (Leu, Ile, Trp, Phe, His, Tyr) exhibited higher median concentrations in children compared to adults in serum. Conversely, lysine and threonine were significantly higher in adult serum. In plasma, age related differences were less pronounced for essential AAs but consistent for specific non-essential AAs. Bubble plot showed Cit, Arg, and Gln were positively associated with age, while GABA was negatively associated with age.

**Conclusion:**

The validated LC–MS/MS platform provides a reliable platform for nutritional biomarker research. Matrix selection and age are critical factors in AA interpretation, offering valuable insights for clinical diagnostics and translational metabolomics research.

## 1 Introduction

Circulating amino acids (AAs) are crucial for understanding nutritional status and detecting metabolic and pathological alterations in tissues (1, 2). Accumulating evidence indicated that AAs played fundamental roles in physiological and pathological processes and are associated with disorders of amino acid metabolism (3), cardiovascular disease (4), cancers (5, 6), healthy aging (7, 8). Therapeutic strategies targeting essential AAs have emerged as a promising area of investigation. For instance, dietary methionine restriction has been shown in murine CRC model to reduce tumor growth and modulated immunotherapy responses by regulating PD-L1 and V-domain Ig suppressor of T cell activation (VISTA) expression (9). AA metabolism regulates the function of effector T cells and regulatory T cells, affecting the function of immune cells. Therefore, investigating AA metabolism in disease contexts may help identify potential metabolic targets for future therapeutic interventions (10). However, epidemiological studies investigating the relationship between methionine exposure and colorectal cancer risk have yielded inconsistent findings. Hospital-based case–control studies have reported an inverse association between methionine intake and CRC risk (11, 12). A meta-analysis of 8 prospective studies involving 431,029 participants and 6,331 colorectal cancer cases reported that higher dietary methionine intake was associated with a lower risk of CRC (13). In contrast, a case–control study in the Chinese population found this inverse association only among women (14).

AAs are classified into essential amino acids (EAAs), which cannot be synthesized *de novo* by the human body, and conditionally essential amino acids, which require supplementation during specific physiological or pathological states. His was an EAA specially for child, and Tyr was considered as conditionally essential amino acids. The remaining AAs, which can be sufficiently synthesized by the human body or converted from other AAs, are termed as non-essential AAs (NEAA). This classification does not imply that nonessential AAs are unimportant. For example, serine, also known as *β*-hydroxyalanine, is a non-essential amino acid that plays a critical role in cellular survival and proliferation. Cells meet this demand through both exogenous serine uptake and glucose re-synthesis (15). Serine metabolism is essential for one-carbon (1C) metabolism, supporting cell growth (16) and carcinogenesis (15, 17). Moreover, serine metabolism has been linked to antitumor immunity, as it may directly influence adaptive immune responses by regulating T-cell proliferation and supplying one-carbon units for *de novo* nucleotide synthesis in proliferating T cells (18). Thus, before translating amino acid-targeted therapies, such as restriction, supplementation, or inhibition of biosynthesis, into clinical practice, robust analytical methods and accurate interpretation of results are essential for precision medicine.

AA concentrations were influenced by multiple factors, including sample preparation (e.g., the coagulation process and anticoagulant type), analytical methods, and individual physiological (e.g., age) and pathological conditions. This study aimed to establish a high-performance liquid chromatography–tandem mass spectrometry (HPLC–MS/MS) method for AA detection, elucidate differences between serum and plasma matrices, and investigate age related variations and correlations. These findings provide a theoretical foundation for interpreting amino acid measurements in both basic and clinical research.

## 2 Methods and materials

### 2.1 Study population

A total of 371 apparently healthy individuals were recruited from the Health Examination Center and Pediatric Health Department of Xinhua Hospital Affiliated to Shanghai Jiao Tong University between January and October 2025. This study included two independent pediatric and adult cohorts. Two hundred thirty-one adults (≥18 years) and 140 children (<18 years), with a subgroup of 26 adults providing paired serum and plasma samples for two matrices comparison and correlation analyses. Plasma specimen enrolled 88 children (median age 9.4 years [6.5–11.7]) and 43 adults (median age 48 years [36–56]). Serum specimen enrolled 52 children (median age 7.8 years [6.8–9.6]) and 188 adults (median age 45 years [37.3–55]).

All participants had laboratory test results within normal ranges (19), including blood count, liver and renal function tests, blood lipid profile, fast glucose. Exclusion criteria included a history of major chronic diseases (e.g., diabetes mellitus, hyperlipidemia, hepatic or renal disease, cardiovascular or cerebrovascular disease, or malignancy), hospitalization within the past 3 months, or current hospitalization. Participants were also excluded if they had recently used amino acid-related supplements or formulations.

### 2.2 Instruments and reagents

The HPLC system was coupled with an API 4500 triple quadrupole mass spectrometer (LC–MS/MS) from AB SCIEX (Massachusetts, USA). Twenty-nine AA standard regents and isotope internal standard regent, calibrators, quality controls, NaOH, and 6-Aminoquinolyl-N-hydroxysccinimidyl carbamate (AQC) were purchased from Zhejiang Diasis Diagnostic Technology Co., Ltd. Formic acid, ammonium acetate, and methanol were acquired from Thermo Fisher Scientific (USA). Deionized water was prepared using a Millipore EQ 7000 water purification system (Millipore Corporation, USA).

### 2.3 Sample process

Collect 2 mL of fasting venous blood from the study subjects using EDTA anticoagulant tubes and/or serum tubes. Centrifuge the samples within 1 h (4,000 r/min, 5 min). Transfer 500 μL of plasma/serum into a 1.5 mL EP tube and store it at −80 °C for subsequent analysis. As per the manufacturer’s protocol, 2.5 μL of the 100× isotopic internal standard was added to 250 μL of methanol to prepare the AA precipitating reagent. Aliquot 50 μL of standard, quality control, and sample solutions into EP tubes were treated with 250 μL precipitating reagent, vortex for 1 min, and centrifuge at 14,000 r/min for 10 min. Transfer 20 μL of the supernatant to a new EP tube and add 70 mL NaOH and 20 μL of 6-Aminoquinolyl-N-hydroxysccinimidyl carbamate (AQC) and derivatize at 50 °C for 10 min. The sample was reconstituted in 200 μL of ultrapure water directly, vortex-mixed, and subsequently analyzed.

### 2.4 Chromatographic and mass spectrometry conditions

Samples were separated on a C18 reversed-phase analytical column (2.6 um, 50 × 4.6 mm, phenomenex, USA) and thermostatically controlled at 40 °C. The mobile phases (MP) used for chromatographic separation were 0.1% CH3COOH/bidistilled water (MP A) and 0.1% CH3COOH/acetonitrile (MP B). The HPLC system was set up with a flow rate of 0.3 mL/min and the following linear gradient in Supplementary Table 1. Positive ion mode was used to obtain the mass spectra (MS1) and the product ion spectra (MS2). The instrument was equipped with a TurboIonSpray source operated at 550 °C and with ion spray voltage of 5,500 V. The biological samples were analyzed with electrospray ionization (ESI), using zero air as nebulizer gas (50 psi) and as heater gas (55 psi). Nitrogen was employed as curtain gas (25 psi) and as collision gas (6 psi) at medium intensity (CAD). The MRM transitions and corresponding mass spectrometric parameters were defined as shown in Supplementary Table 2. Data collection and analysis were performed using Analyst MD 1.7.2 and MultiQuant MD 3.0.3 software, respectively.

### 2.5 Methodology validation

An AQC-based derivatization method was implemented to improve detection sensitivity for amino acids (20). To meet the intended clinical use, the LC–MS/MS method for quantifying 29 AAs was validated for linearity, lower limit of quantitation (LLOQ), precision, accuracy, carryover, and sample stability according to the Clinical and Laboratory Standards Institute (CLSI) approved guideline documents CLSI EP10-A3 (21) and CLSI C62-A (22).

For linearity, six calibrators with concentrations ranging from low to high (specific concentrations are listed in Supplementary Table 3) were reconstituted and processed following the same procedure as actual samples (23). The analysis was performed over three consecutive batches on different days. A linear regression equation was established using the analyte response/isotope internal standard response as the y-value and the analyte ratio (concentration/isotope internal standard concentration) as the *x*-value with weighting factor as 1/*x*^2^. The required correlation coefficient (*R*^2^) for the standard curve was ≥ 0.99 (Supplementary Table 4).

The LLOQ was determined using the lowest concentration calibrator (S1) from the commercial kit. Six replicates were analyzed per batch over three consecutive days. The LLOQ was considered acceptable if the bias was < 20% and the coefficient of variation (CV) was < 20%. Precision was evaluated using quality control (QC) samples QCL (low concentration) and QCH (high concentration) (Supplementary Table 3). Six replicates of each QC level were analyzed per batch. The calculated AA concentrations from the accompanying standard curve were used to determine the within-run precision (intra-assay CV). This process was repeated over three consecutive days to determine the between-run precision (inter-assay CV). The required CV was < 20% for low concentrations near the LLOQ and < 15% for other concentrations, for both within-run and between-run precision. Accuracy was assessed using QC samples QCL and QCH at two concentration levels, processed according to the sample pretreatment protocol. Six replicates were analyzed per batch. The accuracy (measured value/target value × 100%) for each individual measurement within a batch should fall within 85–115% (80–120% for the low concentration point). The mean value of the six replicates per batch should be within ±15% of the target value (±20% for the low concentration point). Carryover was assessed by analyzing a blank sample immediately after analyzing the highest concentration calibrator (S6). This was performed over three consecutive days. The acceptance criterion required that the response of the blank sample for the analyte be less than 20% of the response of S1, and the internal standard response in the blank be less than 5% of the internal standard response in S1.

### 2.6 Statistics

Statistical evaluations were performed using GraphPad Prism (v10.0). Values were presented as the mean ± standard deviation (*x̅* ± *s*) except for special annotation. Student’s *t*-tests were employed to analyze the differences between the groups. For abnormally distributed data, *post-hoc* analyses were performed based on the Tukey test or the Mann–Whitney *U* test. Pearson’s correlation coefficient (normal distributed data), and Spearman’s rank correlation coefficient (non-normal data) were used to assess interrelationships between plasma and serum AA. The sign of correlation coefficient (*r*) indicates the direction of the relationship. Linear regression analysis was subsequently performed to quantify the relationship between amino acid levels in plasma and serum. Deming regression was additionally applied to improve upon ordinary linear regression. Bland–Altman analysis was conducted to evaluate the agreement between the two matrices. A *p*-value < 0.05 was considered statistically significant.

## 3 Results

### 3.1 Methodology validation

In total, 29 AAs were quantified, including 9 EAAs (Leu, Ile, Lys, Met, Phe, Thr, Trp, Val, His) plus 1 conditionally EAA (Tyr), 9 NEAAs (Ala, Arg, Asn, Asp., Glu, Gln, Gly, Pro, and Ser), and 10 amino acid-related metabolites (bAla, Aad, Abu, bAib, Cit, GABA, Hyp, Orn, Sar, and Tau). Linearity, limit of detection (LOD), precision, accuracy, and carryover were successfully validated for each analyte.

All 29 amino acids exhibited good linearity, with correlation coefficients (*R*^2^) of the standard curves exceeding 0.99 (Supplementary Table 4). The maximum bias at the LLOQ was 9.35%, and the maximum CV was 11.20%. Both parameters were within the acceptable limit of 15% (Supplementary Table 4). QC samples at both low (QCL) and high (QCH) concentrations fell within the required ranges. Both the within-run and between-run imprecision (CVs) for all 29 AAs were less than 15% (Supplementary Tables 5, 6). Accuracy at all concentrations was assessed by bias, with values within ± 10% (Supplementary Tables 5, 6). Extraction recovery were also evaluated and demonstrated acceptable reproducibility across all analytes (Supplementary Table 5). Over three consecutive days, the measured values for all 29 AAs in blank samples were less than 20% of the LLOQ response (Supplementary Table 7). No significant carryover was observed for any analyte, with blank responses remaining below predefined acceptance thresholds. These results indicated that the method met acceptable standards for imprecision and trueness. The assay successfully separated all 29 target amino acids within a 10-min chromatographic runtime while maintaining acceptable sensitivity, accuracy, and precision according to predefined validation criteria. The method demonstrated comparable analytical performance in both plasma and serum samples.

### 3.2 Relationship of 29 AAs between plasma and serum

**Figure 1:**
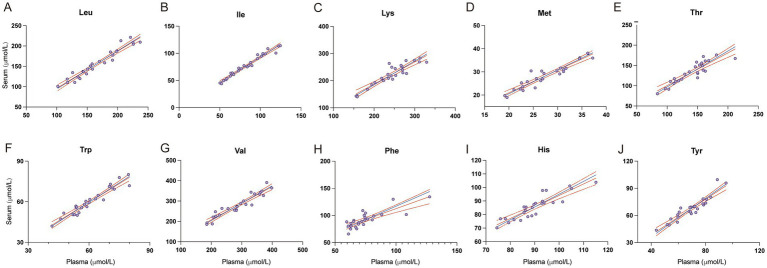
Deming regression analysis of 9 essential amino acids (His for children) and 1 conditionally essential amino acid between plasma and serum. **(A–J)** Each point represents the concentration of a specific amino acid in plasma (*x*-axis) and serum (*y-*axis). The blue solid line represents the Deming regression fit, and the red line indicated the linear regression fit with 95% CI. The Deming regression accounts for measurement errors in both variables. The regression equation were shown on the plot. EAA, essential amino acid; CI, confidence intervals.

We analyzed 26 paired plasma and serum samples to examined relationship of AA levels. The results were grouped by EAAs, NEAAs, and other AAs, and listed in descending order of *r* or R squared (Table 1 and Supplementary Table 8). All of AAs were positive correlated between plasma and serum (Supplementary Table 8). Correlation analysis alone is insufficient to evaluate interchangeability between plasma and serum amino acid concentrations. Linear regression analysis was applied to assess the differences. Fourteen AA showed high correlation (*R*^2^ > 0.85) and 10 showed moderate correlation (0.5 ≤ *R*^2^ ≤ 0.85). Whereas, pronounced concentration disparities were identified for GABA, serine, taurine, sarcosine and asparagine (*R*^2^ < 0.5) (Table 1). Deming regression offered a more appropriate assessment of method agreement than ordinary least-squares regression. The close agreement between Deming (Blue line) and linear regression models (Red line with 95% CI) for most AA indicates that matrix contributed minimally to the observed relationships (Figure 1 and Supplementary Figures 1, 2). Essential amino acids generally exhibited stronger serum/plasma agreement, whereas several NEAAs and other amino acids showed weaker correlations and larger matrix dependent differences.

**Table 1:** Simple linear regression analysis of 29 AAs levels between paired plasma and serum samples from a subgroup of 26 participants.

Type	Amino	Slope	Intercept	95% CI	R squared	*F* value	*p* (two-tailed)
EAA	Ile	0.93	−0.36	0.86 to 1.01	0.9668	699.4	<0.0001
	Leu	0.91	4.98	0.81 to 1.01	0.9372	358.2	<0.0001
	Trp	0.92	4.34	0.81 to 1.04	0.9178	268.0	<0.0001
	Val	0.82	44.10	0.70 to 0.93	0.9015	219.7	<0.0001
	Met	0.97	1.57	0.83 to 1.11	0.8995	214.7	<0.0001
	Lys	0.82	24.08	0.67 to 0.96	0.8497	135.7	<0.0001
	Thr	0.81	19.67	0.64 to 0.97	0.8089	101.6	<0.0001
	His	0.85	9.33	0.67 to 1.03	0.7976	94.57	<0.0001
	Phe	0.80	32.04	0.56 to 1.04	0.6675	48.17	<0.0001
Conditionally essential AA	Tyr	0.99	−1.10	0.83 to 1.15	0.8716	162.9	<0.0001
NEAAs	Gly	0.91	34.22	0.77 to 1.06	0.8749	167.8	<0.0001
	Pro	0.97	−8.01	0.82 to 1.13	0.8706	161.4	<0.0001
	Gln	0.87	89.69	0.72 to 1.03	0.8486	134.5	<0.0001
	Ala	0.91	36.79	0.72 to 1.09	0.8113	103.2	<0.0001
	Asn	1.02	−3.20	0.76 to 1.27	0.7413	68.78	<0.0001
	Glu	0.66	21.12	0.46 to 0.86	0.6526	45.08	<0.0001
	Arg	1.23	62.78	0.82 to 1.66	0.6056	36.85	<0.0001
	Ser	0.49	98.57	0.17 to 0.80	0.2966	10.12	0.004
	Asp	0.07	28.26	−0.35 to 0.49	0.0054	0.129	0.7224
Other AA	bAib	0.97	0.02	0.91 to 1.02	0.9827	1,362	<0.0001
	Abu	0.94	1.22	0.88 to 1.00	0.9772	1,030	<0.0001
	Aad	0.92	0.07	0.86 to 0.99	0.9705	790.7	<0.0001
	Hyp	1.25	0.51	1.15 to 1.36	0.9628	621.8	<0.0001
	bAla	1.17	−1.09	1.03 to 1.30	0.93	318.7	<0.0001
	Cit	0.93	1.22	0.81 to 1.06	0.9089	239.4	<0.0001
	Orn	0.54	22.15	0.37 to 0.71	0.6432	43.26	<0.0001
	GABA	0.53	0.05	0.27 to 0.78	0.4365	18.59	0.0002
	Tau	0.62	23.92	0.07 to 1.18	0.1826	5.363	0.0294
	Sar	0.05	0.38	0.003 to 0.105	0.1678	4.838	0.0377

Categorized by AA types and strength of relationship. EAA, essential amino acid; NEAA, non-essential amino acid; Ala, alanine; Arg, arginine; Asn, asparagine; Asp., aspartate; Cit, citrulline; Gln, glutamine; Glu, glutamate; Gly, glycine; His, histidine; Hyp, hydroxyproline; Ile, isoleucine; Leu, leucine; Lys, lysine; Met, methionine; Orn, ornithine; Phe, phenylalanine; Pro, proline; Sar, sarcosine; Ser, serine; Tau, taurine; Thr, threonine; Trp, tryptophan; Tyr, tyrosine; Val, valine; bAla, *β*-alanine; Aad, *α*-aminoadipic acid; Abu, *α*-aminobutyric acid; bAib, *β*-aminoisobutyric acid; GABA, *γ*-aminobutyric acid.

**Figure 2:**
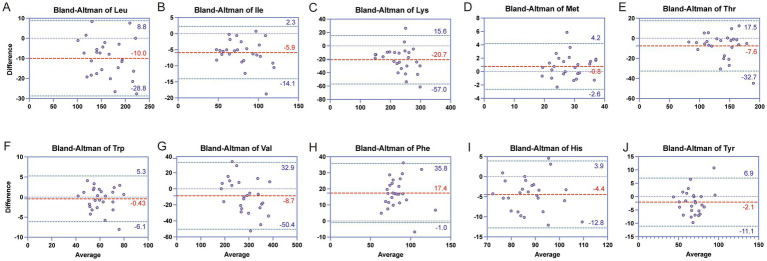
Bland–Altman agreement analysis of plasma and serum concentrations for 9 essential amino acids (His for children) and 1 conditionally essential amino acid. Scatter points represented 26 individual sample data. **(A–J)** The *x-*axis of each point denoted the average value of paired measurements from the two matrices per sample, while the *y* axis represented the difference between the paired measurement values of the two matrices per sample. The light blue dotted lines indicated the 95% Limits of Agreement (95% LoA), and the red dashed line marked the mean difference of measurements obtained from the two testing approaches.

To further evaluate the agreement between serum and plasma amino acid measurements, Bland–Altman analyses were performed for AA. The majority of observations fell within the 95% limits of agreement, indicating acceptable concordance between the two matrices (Figure 2 and Supplementary Figures 3, 4). However, systematic biases were observed for several amino acids. Take EAA as an example, Leu, Ile, Lys, and Val exhibited negative mean biases, indicating generally lower concentrations in serum relative to plasma. Among these amino acids, Lys and Val showed the largest absolute biases and widest limits of agreement, suggesting substantial matrix dependent variability. In contrast, Phe demonstrated a positive mean bias, indicating consistently higher concentrations in serum than in plasma, whereas Trp showed minimal bias and the narrowest limits of agreement, reflecting excellent agreement between the two matrices. Collectively, these findings suggest that although serum and plasma measurements were broadly comparable for several essential amino acids, matrix dependent systematic differences existed for specific analytes and should be considered when interpreting amino acid profiles.

### 3.3 Differences in amino acid measurements between serum and plasma samples from pediatric and adult cohorts

Serum and plasma exhibited systematic differences in the concentrations of several amino acids, potentially reflecting biochemical changes associated with the coagulation process (24) and differential metabolite release or consumption during sample processing. Twenty-nine amino acids from serum and plasma samples from non-overlapping individuals were analyzed by LC–MS/MS (Table 2). As shown in Supplementary Figure 5D, overall, 6 of the 29 AA (Sar, GABA, Orn, Tau, Asn, and bAla) were higher in plasma, whereas other 10 AA (Asp., Arg, Phe, Ser, Gly, Met, Trp, Gln, Val, and Glu) were higher in serum (*p* < 0.05, *q* < 0.01). 13 of 29 AAs were equal between serum and plasma (Supplementary Table 9 and Supplementary Figure 5). Results were listed in descending order according to −log10 (*q* value).

**Table 2:** Concentrations of 29 amino acids in plasma and serum of children (<18 years) and adults (≥18 years).

Type of AA	Amino	Plasma		Plasma		Serum		Serum	
		<18 years (*n* = 88)		≥18 years (*n* = 43)		<18 years (*n* = 52)		≥18 years (*n* = 188)	
		Mean	SD	Mean	SD	Mean	SD	Mean	SD
Age, y*		9.7	6.5–11.7	48	36.0–56.0	7.8	6.8–9.6	45	37.3–55
EAA	Phe	69.05	12.73	74.09	13.47	128.1	21.6	91.87	14.42
	Trp	54.7	9.51	59.35	8.91	65.06	13.65	59.72	9.373
	Val	265.6	53.64	279.1	57.69	297.1	72.2	288.4	49.62
	Thr	140.6	29.2	147.7	36.32	135.3	30.63	151	34.14
	Met	25.86	6.759	27.61	5.285	31.28	9.657	29.3	4.865
	Lys	212.1	37.72	239.9	41.05	196.4	52.84	231.2	41.4
	Leu	153.2	31.35	161.1	33.25	183.0	55.74	156.4	29.05
	Ile	81.13	20.62	81.12	19.31	94.96	34.72	80.83	16.94
Children specific EAA	His	90.27	10.16	91.75	10.47	99.02	13.18	90.99	11.66
Conditionally essential AA	Tyr	78.23	15.89	69.85	11.46	84.24	21.45	71.3	13.28
NEAA	Ala	440.8	102	459.7	131	445.3	112.6	455.6	108.8
	Glu	93.4	20.24	90.78	24.04	174.6	50.09	87.79	23.29
	Arg	34.17	13.7	42.83	16.84	130.3	33.01	117.9	27.02
	Gly	271.4	53.71	286.7	73	366.1	66.77	306.9	61.12
	Ser	160	26.44	152.2	24.4	215	32.43	177.7	24.77
	Pro	208.8	58.25	212.9	73.88	200.8	60.61	204.5	59.73
	Asp	9.519	2.393	8.442	2.967	33.99	9.916	29.12	5.71
	Asn	48.85	8.251	52.39	7.535	46.53	10.13	45.36	6.669
	Gln	485.4	60.62	532.1	66.83	439.9	76.41	559.7	72.32
Other AA	Orn	142.1	32.61	160.4	44.9	81.47	26.17	119.7	30.75
	Tau	134.7	39.19	150.9	26.65	90.71	41.67	117.1	33.16
	Hyp	22.02	8.06	11.79	5.677	28.1	10.37	17.01	9.312
	Cit	29.2	5.32	32.58	7.758	28.72	7.32	32.1	7.394
	Sar	6.87	2.36	19.62	6.97	1.98	0.72	1.51	0.50
	bAib	1.9	1.252	2.034	1.515	1.624	1.07	1.996	1.45
	Abu	24.48	7.879	23.34	7.818	25.92	8.842	25.42	8.333
	bAla	4.131	1.142	3.606	1.278	3.933	2.277	3.361	1.103
	Aad	0.9759	0.3851	0.9919	0.4521	1.175	0.6567	0.9588	0.3954
	GABA	0.4142	0.1137	0.3302	0.08645	0.3355	0.118	0.2241	0.04185

^*^Data was presented as Median [Q1–Q3]. Results of children and adults AA were obtained from independent cohorts. EAA, essential amino acid; NEAA, non-essential amino acid; Ala, alanine; Arg, arginine; Asn, asparagine; Asp., aspartate; Cit, citrulline; Gln, glutamine; Glu, glutamate; Gly, glycine; His, histidine; Hyp, hydroxyproline; Ile, isoleucine; Leu, leucine; Lys, lysine; Met, methionine; Orn, ornithine; Phe, phenylalanine; Pro, proline; Sar, sarcosine; Ser, serine; Tau, taurine; Thr, threonine; Trp, tryptophan; Tyr, tyrosine; Val, valine; bAla, β-alanine; Aad, α-aminoadipic acid; Abu, α-aminobutyric acid; bAib, β-aminoisobutyric acid; GABA, γ-aminobutyric acid.

**Figure 3:**
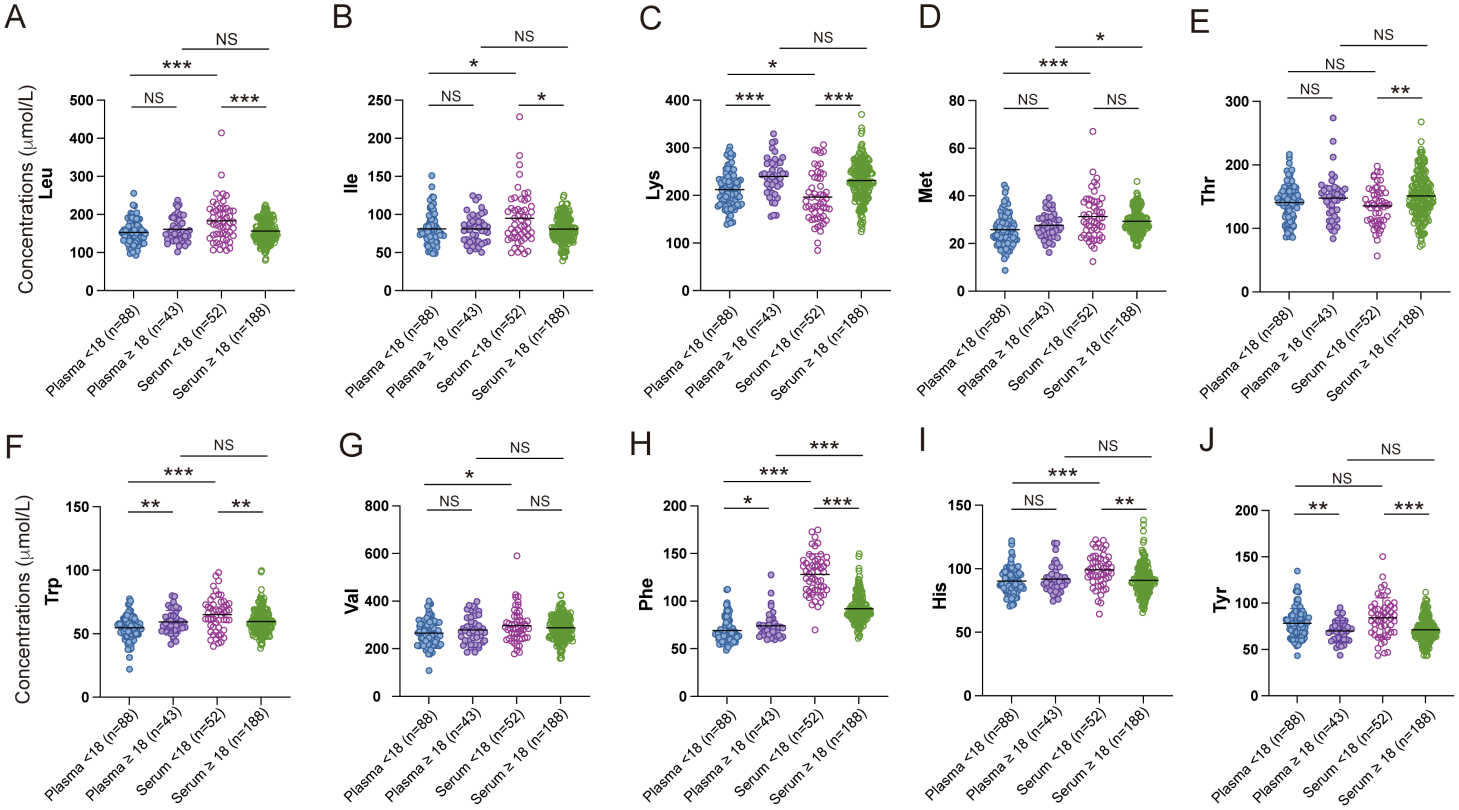
The expression profiles of the 9 essential amino acids (His for children) and 1 additionally essential amino acid across different age groups and samples. **(A–J)** Solid circles represent plasma results, while open circles represent serum results. Comparisons between children and adults were performed using independent cohorts. Statistically significant showed as *p < 0.05, **p < 0.01, ***p < 0.001.

We further investigated differences between adults and children. Most EAAs levels of children (Try, Val, Met, His, Phe, Leu, and Ile) were significantly lower in plasma than in serum, while lysine was higher in plasma. No significant difference in Thr concentrations was observed between plasma and serum in children. In adults, concentrations were nearly equal between plasma and serum, except for Met and Phe (Figure 3). Conversely, the concentrations of 3 non-essential AAs (Ala, Pro and Gln) were generally comparable between plasma and serum. Arg, Ser, and Asp were more abundant in serum in both children and adults (Supplementary Figure 6). Asn was higher in adult plasma, while Gly and Glu were higher in children’s serum (Supplementary Figure 6). Among the remaining nine amino acids, Orn, Tau, Hyp, and GABA showed significant differences in both plasma and serum between age groups (<18 years vs. ≥18 years). Except for Hyp, Orn, Tau and GABA were present at higher concentrations in plasma than in serum. The other five AA showed no significant changes (Supplementary Figure 7). These differences may influence the interpretation and comparability of amino acid measurements.

### 3.4 Age dependent amino acid profiles

Several studies have reported differences in amino acid profiles between children and adults across various physiological and metabolic dimensions (25, 26). These differences may be influenced by distinct stages of growth and development, physiological demands, hormonal levels, and potential pathological conditions. We performed an in-depth analysis of 29 AAs in adults (≥18 years) and children (<18 years). Most AA concentrations differed significantly between adults and children, although the magnitude of these differences varied between serum and plasma (Table 2). EAAs like Leu, Ile, Met, Trp, Phe, His, and Tyr showed higher median serum concentrations in children than in adults, with distributions showing an overall upward shift rather than being driven by outliers. In contrast, Lys and Thr were significantly higher in adult serum. No obvious age related difference was observed for Val in serum (Figure 3). In plasma, most EAAs (Leu, Ile, Met, Thr, Val, His, and Tyr) showed no significant differences between adults and children. Plasma levels of Lys, Trp, and Phe were higher in adults than in children, whereas Tyr levels were higher in children than in adults (Figure 3).

For the nine non-essential AAs in serum, Gly, Glu, Arg, Ser, and Asp displayed markedly higher levels in children than in adults, whereas Gln was lower in children than in adults. The median serum concentrations of Ala, Pro, and Asn were comparable between adults and children. These AAs are known to participate in diverse physiological processes, including protein synthesis, redox regulation, and nitrogen metabolism, which may contribute to their relatively stable circulating concentrations (Supplementary Figure 6). In plasma, age related patterns of non-essential amino acids were similar to those of EAAs. Most non-essential AAs showed comparable levels between adults and children (Ala, Pro, Gly, Glu, and Ser), or only modest changes (Asp and Asn). Plasma Gln level was also lower in children than in adults, and plasma Asp concentrations decreased with age (Supplementary Figure 6). Among the remaining nine AAs, serum levels of β-Ala, Aad, Hyp, and GABA were overall higher in children than in adults. In both serum and plasma, Cit, Orn, and Tau were higher in adults than in children (Supplementary Figure 7). Plasma levels of β-Ala, Hyp, and GABA were also higher in children, while Aad showed no age related difference in plasma. bAib and Abu showed no significant differences between adults and children in either serum or plasma (Supplementary Figure 7).

**Figure 4:**
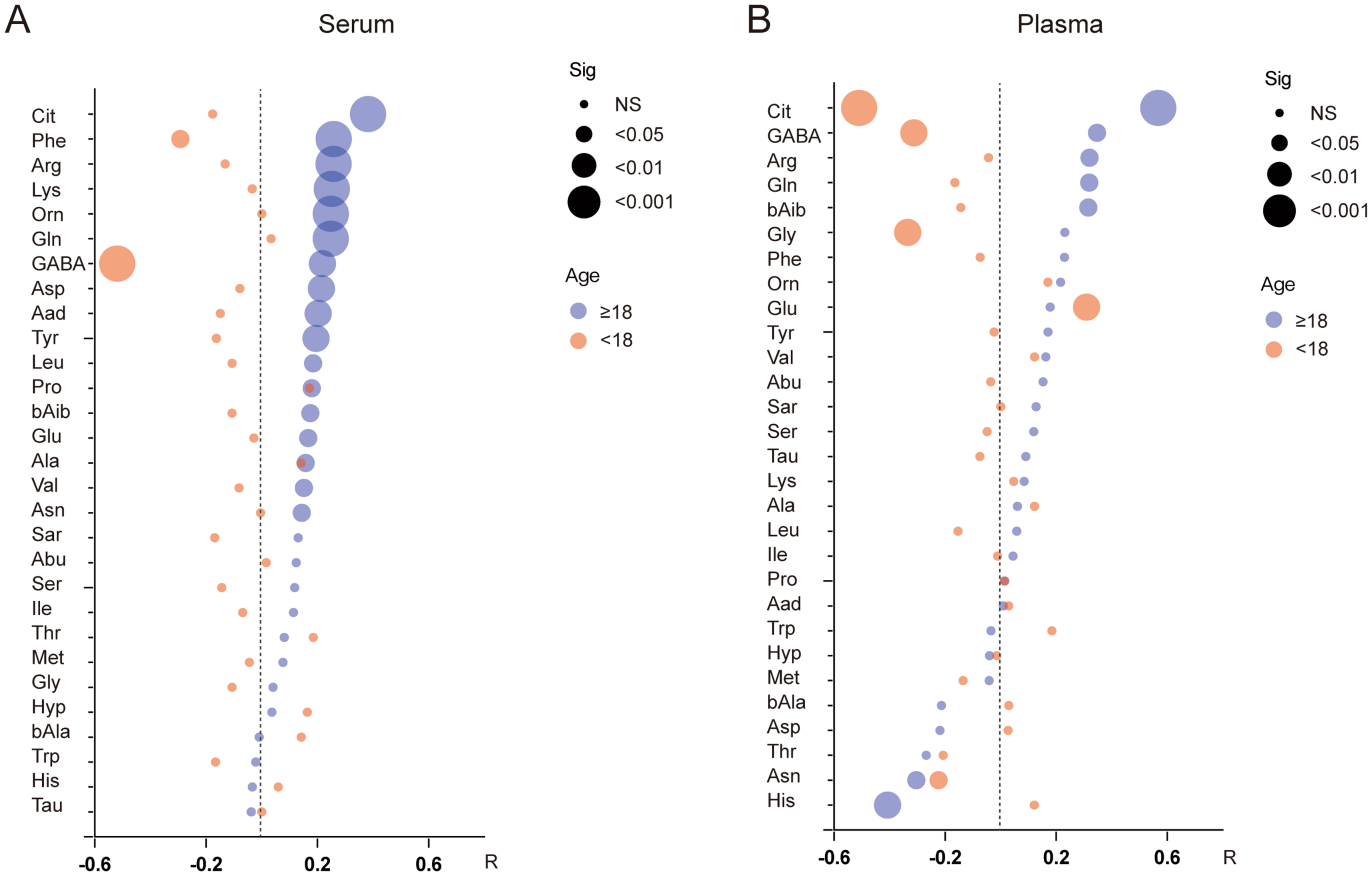
Bubble plot analysis of age associated amino acids in serum and plasma. Analysis between children and adults were performed using independent cohorts. Bubble plots illustrate the associations between age and the concentrations of 29 amino acids measured in serum and plasma. Each bubble represents one amino acid. The direction of association with age is indicated by *x*-axis. Adult subjects (≥18 years) was defined as blue bubble, while children (<18 years) was defined as orange bubble. Statistical significance of the association between aminos and age effect is denoted by bubble size reflects, and ns indicating no significant association. **(A)** In serum, a broad range of amino acids shows strong age related associations. **(B)** In contrast, age related effects in plasma are attenuated and restricted to a smaller subset of amino acids, suggesting tighter short-term metabolic homeostasis in circulating plasma. Ala, alanine; Arg, arginine; Asn, asparagine; Asp., aspartate; Cit, citrulline; Gln, glutamine; Glu, glutamate; Gly, glycine; His, histidine; Hyp, hydroxyproline; Ile, isoleucine; Leu, leucine; Lys, lysine; Met, methionine; Orn, ornithine; Phe, phenylalanine; Pro, proline; Sar, sarcosine; Ser, serine; Tau, taurine; Thr, threonine; Trp, tryptophan; Tyr, tyrosine; Val, valine; bAla, *β*-alanine; Aad, *α*-aminoadipic acid; Abu, *α*-aminobutyric acid; bAib, *β*-aminoisobutyric acid; GABA, *γ*-aminobutyric acid.

Finally, we explored the relationship between serum and plasma concentrations of 29 amino acids and chronological age. Bubble plot analysis revealed that age associated AAs were more prominent in serum than in plasma. A broad range of amino acids (Cit, Arg and Gln as top three) exhibited positive associations with age, while GABA was negatively correlated with age both in serum and plasma (Figure 4). In plasma, negative associations were observed for His and Asn. These findings indicated that circulating amino acid profiles varied with age and that several amino acids exhibited significant associations with chronological age.

## 4 Discussion

AA analyses can be performed using either plasma or serum (27, 28). Both matrices were widely used in clinical research, thereby advancing diagnostics, disease monitoring, therapy prediction, and nutritional interventions (29, 30). However, several metabolites showed statistically significant alterations among the blood matrices, and their correlations are affected (31). These differences can directly impact the results of AA testing. In this study, we developed and validated a rapid LC–MS/MS method for the simultaneous quantification of 29 amino acids in human blood samples. Our results confirmed significant matrix dependent concentration differences for several analytes. Furthermore, distinct age related AA profiles were identified, with children exhibiting higher concentrations of several essential amino acids. Notably, serum may be more sensitive than plasma for detecting certain age related differences in amino acid profiles, suggesting potential implications for biomarker matrix selection. Together, these findings highlight the importance of specimen selection in AA profiling and provide new insights into age related metabolic variation in circulating amino acids.

Approximately 2% of AAs exist in the form of free form in the human body. The rest remainder incorporated into proteins. The ratio of free to protein-bound AAs varies greatly across different amino acids and among various tissues. Generally, essential amino acids constitute a smaller proportion compared to non-essential amino acids (32). AAs are transported in blood plasma, and blood cells also contain significant amounts of certain AAs. For instance, aspartic acid is predominantly transported by red blood cells, resulting in its very low concentration in serum or plasma. Glycine exhibits a much higher intracellular concentration than its plasma level (33). Free AA concentrations are higher in serum, which could potentially facilitate the detection of age related dynamic changes. In plasma, binding of AAs to coagulation factors may lead to an apparent overestimation of certain AA levels. Key metabolic markers such as Cit, GABA, and Gly show consistent age related associations in both sample types, indicating that their biological relevance is independent of technical artifacts. Most amino acids demonstrated acceptable agreement between serum and plasma, with Deming regression closely matching ordinary linear regression and no evidence of substantial proportional bias. Nevertheless, several AA like Lys, Val, Thr, and Phe exhibited greater matrix dependent variability, indicating that serum and plasma should not be considered fully interchangeable for all amino acid measurements. The observed specimen matrix differences may reflect metabolic alterations occurring during clot formation. An important consideration when interpreting the present findings was that results were obtained from a subset of 26 adult participants with paired serum and plasma samples. Consequently, the most robust assessment of matrix effects was derived from these paired analyses. In contrast, comparisons involving the broader pediatric and adult cohorts were used independent populations and may therefore reflect a combination of age related, biological, and sampling related differences in addition to potential matrix effects.

Our results highlighted the age related differences in AA profiles as well. During rapid growth and development, children have a higher demand for AAs, particularly for essential AAs. In both serum and plasma, the number of essential AAs enriched in children was markedly greater than those enriched in adults, reflecting the systemic demand for essential amino acids during growth and development. In contrast, Lys and Thr were serum specific examples of amino acids more abundant in adults. Unlike EAAs, non-essential AAs more directly reflect endogenous synthesis, energy metabolism, and nitrogen flux. Consistent with EAAs, serum non-essential AA levels exhibited more pronounced age related differences than plasma levels. Within serum samples, most non-essential AAs were significantly higher in children than in adults, indicating an age dependent metabolic profile associated with growth and increased nitrogen turnover. In contrast, age related differences in plasma were limited and observed only for a small subset of AAs, suggesting tighter short-term metabolic regulation in circulating plasma.

Few studies have explored the relationship between amino acid profiles and healthy aging (8). Beyond the differences observed between children and adults, several amino acids exhibited significant associations with chronological age. In particular, Cit, Arg, and Glu showed positive associations with age, whereas GABA demonstrated a consistent negative association in both serum and plasma. Citrulline and arginine are key intermediates in nitrogen metabolism and nitric oxide synthesis (34), while glutamine plays an essential role in nitrogen transport, immune function, and cellular metabolism (34). The observed age related increases in these metabolites may therefore reflect physiological adaptations occurring throughout adulthood. In contrast, the decline in GABA with age may indicate alterations in metabolic or neuroendocrine regulation (35). Although the biological significance of these changes remains incompletely understood, our findings are consistent with the concept that circulating amino acid profiles undergo measurable age associated remodeling and may provide useful insights into age related metabolic variation.

However, relevant research evidence remains limited and inconsistent. Taurine provides an illustrative example of the complexity of age related amino acid changes. As a conditionally essential nutrient involved in osmoregulation, antioxidative defense, bile acid conjugation, and mitochondrial function, taurine has attracted considerable attention in aging research. Few studies have found that circulating taurine concentrations decrease with advancing age and indicated that taurine supplementation can ameliorate various age related phenotypes and extend the lifespan of model organisms (36). However, Cohort (37) and experimental evidence (38) reported that the association between circulating taurine levels and functional indicators of health status is context-dependent (cohort or age), suggesting that low circulating taurine is unlikely to serve as a robust biomarker of aging. This phenomenon was observed in both longitudinal and cross-sectional analyses, suggesting that changes in free taurine concentration are not universal features of aging. Our results demonstrated that Tau in both plasma and serum were higher in adults than in children, yet no significant positive correlation with age was observed (Supplementary Figure 8). Given that many studies do not explicitly specify the biological specimen analyzed, it is plausible that all reported findings are valid, as the sample type may act as a confounding factor affecting AA measurements. Furthermore, confounding factors such as platelets present in plasma samples may further obscure age related differences, making serum a more suitable matrix for detecting age related changes in AA profiles.

This study has several important strengths. Our study integrated analytical validation with clinically relevant biological applications. Rather than focusing solely on method development, the investigation explored how specimen type and age influence amino acid measurements, thereby enhancing the translational relevance of the findings. First, a comprehensive LC–MS/MS method was applied for the simultaneous quantification of 29 amino acids. The assay demonstrated excellent analytical performance, including satisfactory linearity, precision, accuracy, sensitivity, and minimal carryover across all analytes. Furthermore, all target compounds were successfully separated within a short chromatographic runtime, supporting the feasibility of high-throughput amino acid profiling in both clinical and population-based studies. Such analytical robustness provides a reliable foundation for subsequent biological and epidemiological investigations. Second, this study systematically evaluated differences between serum and plasma, two of the most commonly used matrices for amino acid testing. Although serum and plasma were often used interchangeably in clinical practice and research, direct comparisons across a broad panel of amino acids remained limited. By examining both correlation patterns and concentration differences, this study demonstrated that while most amino acids showed moderate to strong correspondence between matrices, several analytes exhibited substantial matrix dependent variation. These findings provided practical guidance for specimen selection and contribute to the standardization of amino acid measurements in nutritional and metabolic studies. Third, the inclusion of both pediatric and adult participants enabled a comprehensive assessment of age related amino acid profiles. The study identified distinct patterns among essential amino acids, non-essential amino acids, and amino acid-related metabolites, highlighting the influence of developmental stage on circulating amino acid concentrations. These findings provided valuable reference information for future studies investigating nutritional status, metabolic health, and age related physiological changes.

Several limitations should also be considered when interpreting the findings. First, the comparison between serum and plasma was based on a moderate number of paired samples. Although these paired samples permitted direct evaluation of matrix differences, most comparisons between serum and plasma were derived from independent participant groups. Accordingly, matrix dependent conclusions should be interpreted primarily in the context of the paired sample analyses. Though statistically significant differences were identified for multiple amino acids, the modest sample size may limit the precision of estimated matrix effects and reduce the generalizability of the findings. Larger validation cohorts are warranted to confirm the observed serum/plasma differences and establish matrix-specific reference intervals. Second, the study employed a cross-sectional design, which limits the ability to infer causal relationships between age and amino acid concentrations. The observed differences between children and adults may reflect a combination of developmental, physiological, dietary, and lifestyle factors rather than age alone. Longitudinal studies are needed to characterize dynamic changes in amino acid metabolism across the life course and to better understand the biological mechanisms underlying age related variations. Third, potential sex-specific differences in amino acid profiles were not systematically evaluated. Given the known influence of sex hormones, body composition, and metabolic regulation on amino acid metabolism, future studies with larger and more balanced populations are warranted to investigate sex-dependent variations in circulating amino acid concentrations. Finally, although significant differences between serum and plasma were identified for several amino acids, the study primarily relied on correlation and concentration comparisons. Additional age stratified analyses may provide a more comprehensive evaluation of the interchangeability of serum and plasma measurements. Furthermore, mechanistic investigations are required to clarify the biological processes responsible for matrix-dependent differences, including the potential contributions of coagulation related metabolism, platelet activation, and cellular release of amino acids during sample processing.

In conclusion, we established and validated a rapid and reliable LC–MS/MS method for the simultaneous quantification of 29 amino acids and amino acid-related metabolites in human blood samples. The observed matrix dependent differences suggested that plasma and serum should not be considered interchangeable for all amino acids. Consequently, matrix specific reference values and standardized specimen collection procedures may be necessary when amino acid profiles are used as nutritional or metabolic biomarkers. Future studies involving larger and more diverse populations, longitudinal follow-up, and mechanistic investigations are warranted to further elucidate the biological and clinical significance of age related amino acid variation and to support the development of robust amino acid-based biomarkers for nutritional and metabolic health assessment.

## Data Availability

The raw data supporting the conclusions of this article will be made available by the authors, without undue reservation.
