# Supplementary material for: Comparative profiling of amino acids in plasma and serum across children and adults using LC–MS/MS and its implications for precision nutrition
Source: Front Nutr. 2026 Jul 24;13:1850080. doi: 10.3389/fnut.2026.1850080 (PMC13447054; doi:10.3389/fnut.2026.1850080)
Supplement: Supplementary file 1 [file Data_Sheet_1.pdf]

## Supplementary Material

# 1 Supplementary Figures and Tables

## 1.1 Supplementary Figures 1

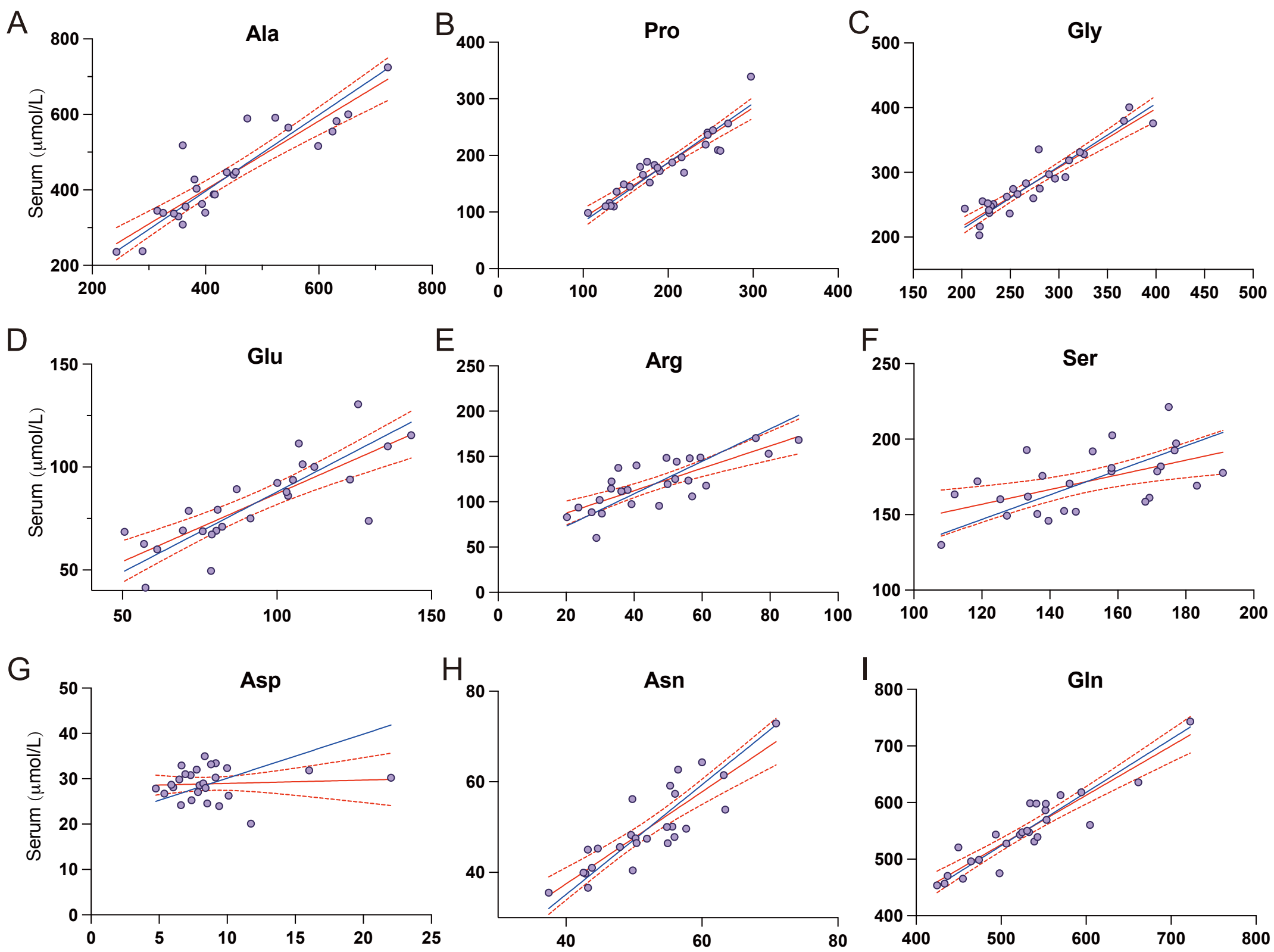


**SFigure 1.** Deming regression analysis of 9 non-essential amino acids between plasma and serum. Each point represents the concentration of a specific amino acid in plasma (X axis) and serum (Y axis). The blue solid line represents the Deming regression fit, and the red line indicated the linear regression fit with 95% CI. The Deming regression accounts for measurement errors in both variables. The regression equation were shown on the plot. CI, confidence intervals; Ala, alanine; Arg, arginine; Asn, asparagine; Asp, aspartate; Gln, glutamine; Glu, glutamate; Gly, glycine; Pro, proline; Ser, serine.

## 1.2 Supplementary Figures 2

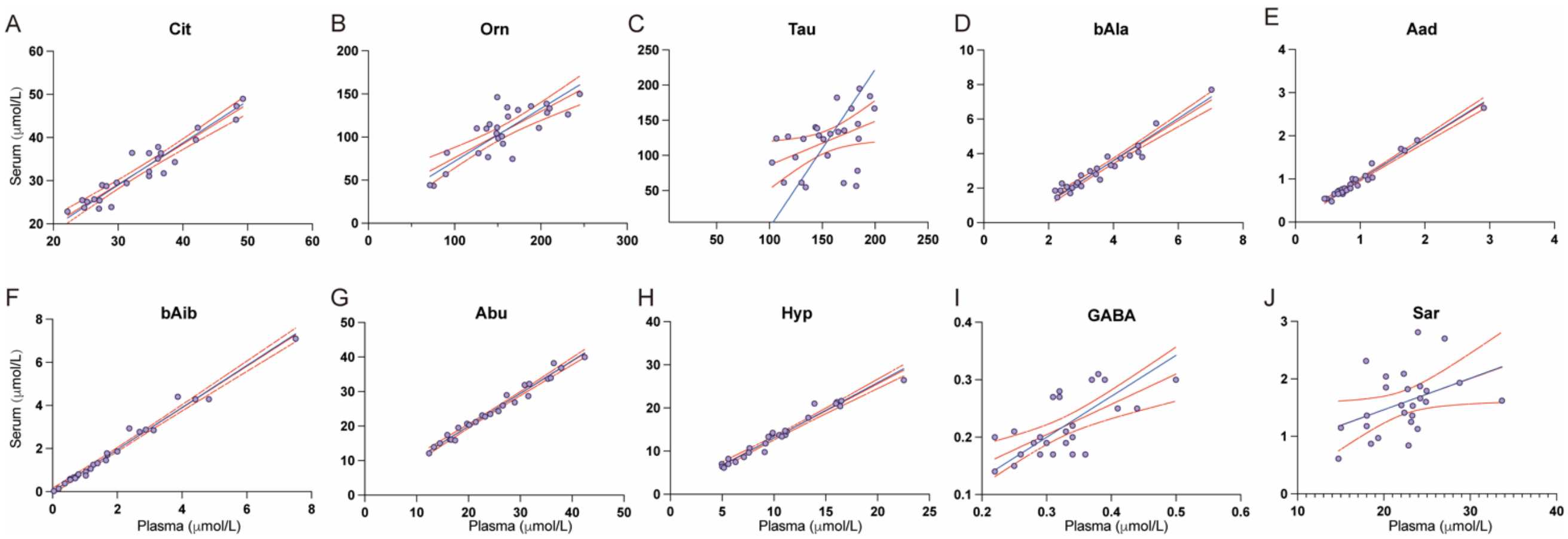


**SFigure 2.** Deming regression analysis of 10 other amino acids between plasma and serum. Each point represents the concentration of a specific amino acid in plasma (X axis) and serum (Y axis). The blue solid line represents the Deming regression fit, and the red line indicated the linear regression fit with 95% CI. The Deming regression accounts for measurement errors in both variables. The regression equation were shown on the plot. CI**,** confidence intervals**;** Cit, citrulline; Hyp, hydroxyproline; Orn, ornithine; Sar, sarcosine; Tau, taurine; bAla, β-alanine; Aad, α-aminoadipic acid; Abu, α-aminobutyric acid; bAib, β-aminoisobutyric acid; GABA, γ-aminobutyric acid.

## 1.3 Supplementary Figures 3

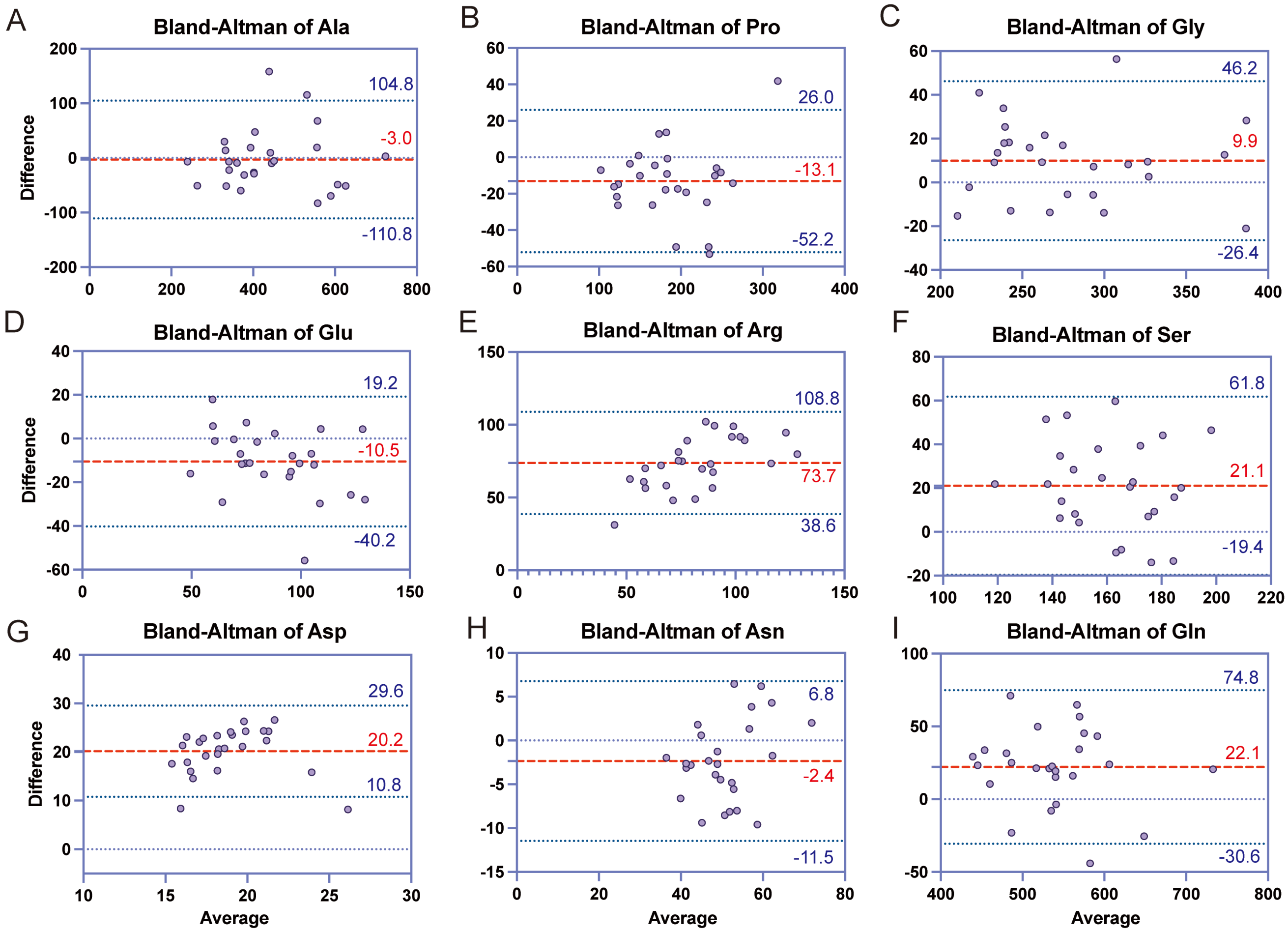


**SFigure 3.** Bland-Altman agreement analysis of plasma and serum concentrations for 9 non-essential amino acids. Scatter points represented 26 individual sample data. The x axis of each point denoted the average value of paired measurements from the two matrices per sample, while the y axis represented the difference between the paired measurement values of the two matrices per sample. The light blue dotted lines indicated the 95% Limits of Agreement (95% LoA), and the red dashed line marked the mean difference of measurements obtained from the two testing approaches. EAA, essential amino acid; CI, confidence intervals; Ala, alanine; Arg, arginine; Asn, asparagine; Asp, aspartate; Gln, glutamine; Glu, glutamate; Gly, glycine; Pro, proline; Ser, serine.

## 1.4 Supplementary Figures 4

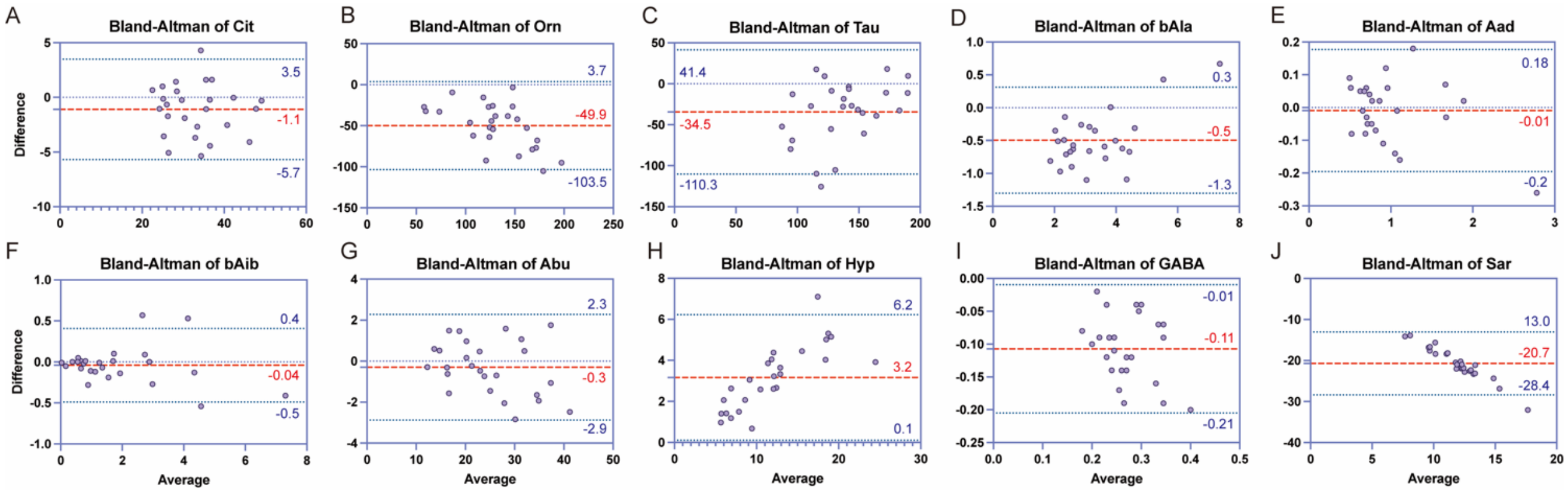


**SFigure 4.** Bland-Altman agreement analysis of plasma and serum concentrations for 10 other amino acids. Scatter points represented 26 individual sample data. The x axis of each point denoted the average value of paired measurements from the two matrices per sample, while the y axis represented the difference between the paired measurement values of the two matrices per sample. The light blue dotted lines indicated the 95% Limits of Agreement (95% LoA), and the red dashed line marked the mean difference of measurements obtained from the two testing approaches. Cit, citrulline; Hyp, hydroxyproline; Orn, ornithine; Sar, sarcosine; Tau, taurine; bAla, β-alanine; Aad, α-aminoadipic acid; Abu, α-aminobutyric acid; bAib, β-aminoisobutyric acid; GABA, γ-aminobutyric acid.

## 1.5 Supplementary Figures 5

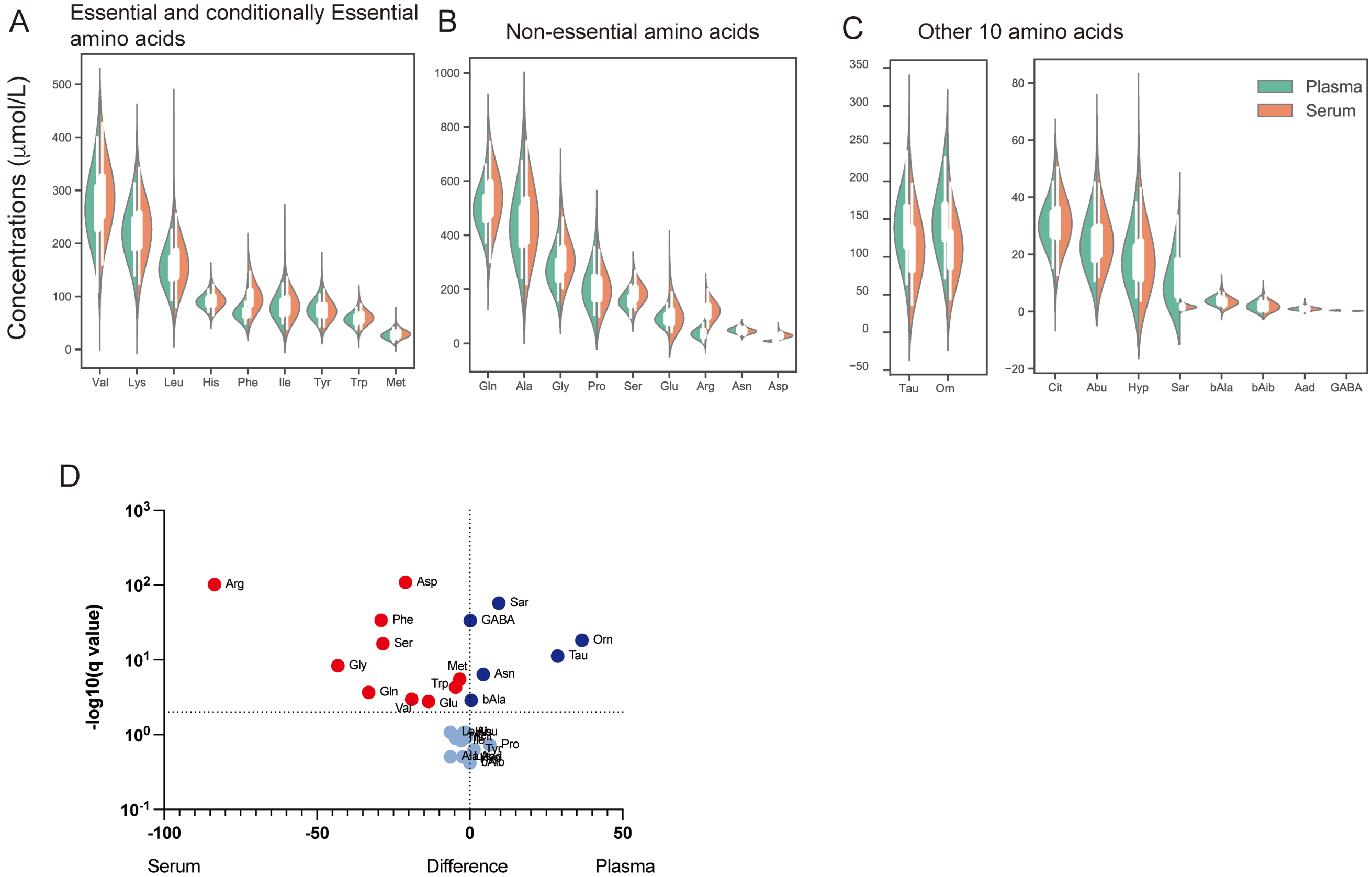


**SFigure 5.** Comparison of the concentrations of 29 amino acids in serum and plasma. (A) Violin plots of 9 essential amino acids in serum and plasma of participants. (B) Violin plots of 9 non-essential amino acids in serum and plasma. (C) Violin plots of 10 other amino acids in serum and plasma. (D) The volcano plot reveals differentially abundant amino acids in serum and plasma. Blue dots represented amino acids elevated in plasma, while red dots indicated amino acids elevated in serum. Abbr: EAA, essential amino acid; NEAA, non-essential amino acid; Ala, alanine; Arg, arginine; Asn, asparagine; Asp, aspartate; Cit, citrulline; Gln, glutamine; Glu, glutamate; Gly, glycine; His, histidine; Hyp, hydroxyproline; Ile, isoleucine; Leu, leucine; Lys, lysine; Met, methionine; Orn, ornithine; Phe, phenylalanine; Pro, proline; Sar, sarcosine; Ser, serine; Tau, taurine; Thr, threonine; Trp, tryptophan; Tyr, tyrosine; Val, valine; bAla, β-alanine; Aad, α-aminoadipic acid; Abu, α-aminobutyric acid; bAib, β-aminoisobutyric acid; GABA, γ-aminobutyric acid.

## 1.6 Supplementary Figures 6

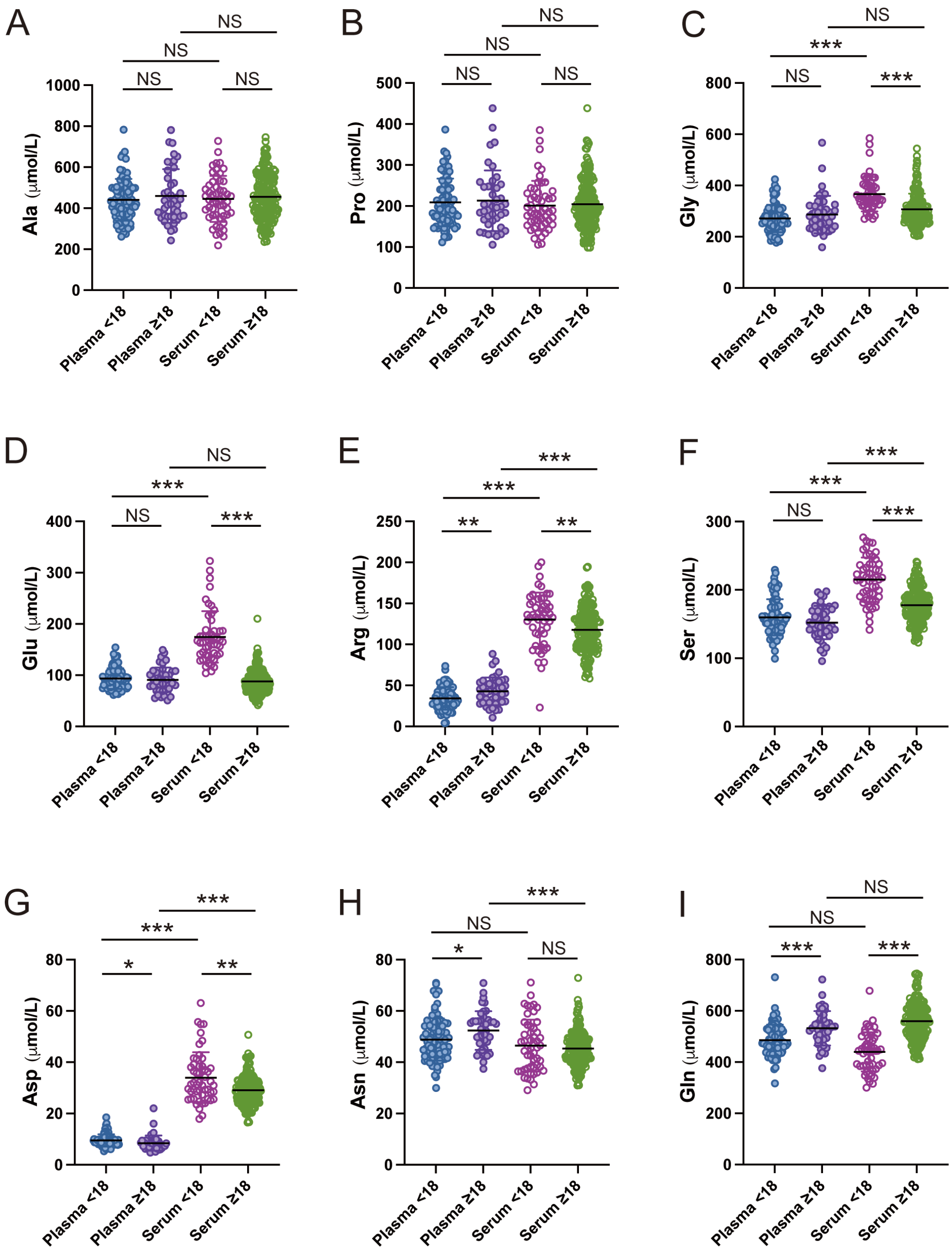


**SFigure 6.** The expression profiles of the 9 non-essential amino acids across different age groups and samples. Solid circles represent plasma results, while open circles represent serum results. EAA, essential amino acid; CI, confidence intervals; Ala, alanine; Arg, arginine; Asn, asparagine; Asp, aspartate; Gln, glutamine; Glu, glutamate; Gly, glycine; Pro, proline; Ser, serine.

## 1.7 Supplementary Figures 7

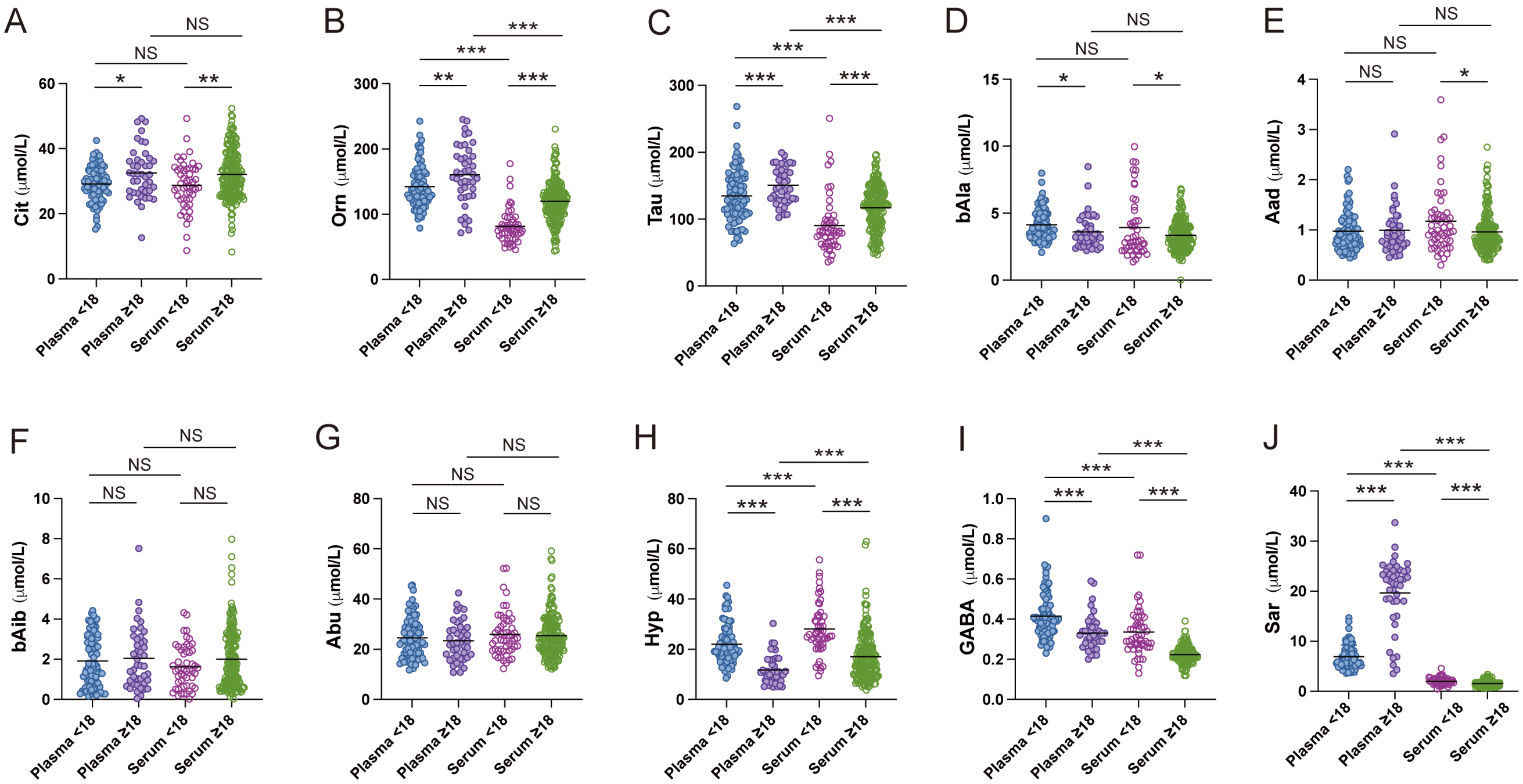


**SFigure 7.** The expression profiles of other 10 amino acids across different age groups and samples. Solid circles represent plasma results, while open circles represent serum results. Cit, citrulline; Hyp, hydroxyproline; Orn, ornithine; Sar, sarcosine; Tau, taurine; bAla, β-alanine; Aad, α-aminoadipic acid; Abu, α-aminobutyric acid; bAib, β-aminoisobutyric acid; GABA, γ-aminobutyric acid.

## 1.8 Supplementary Figures 8

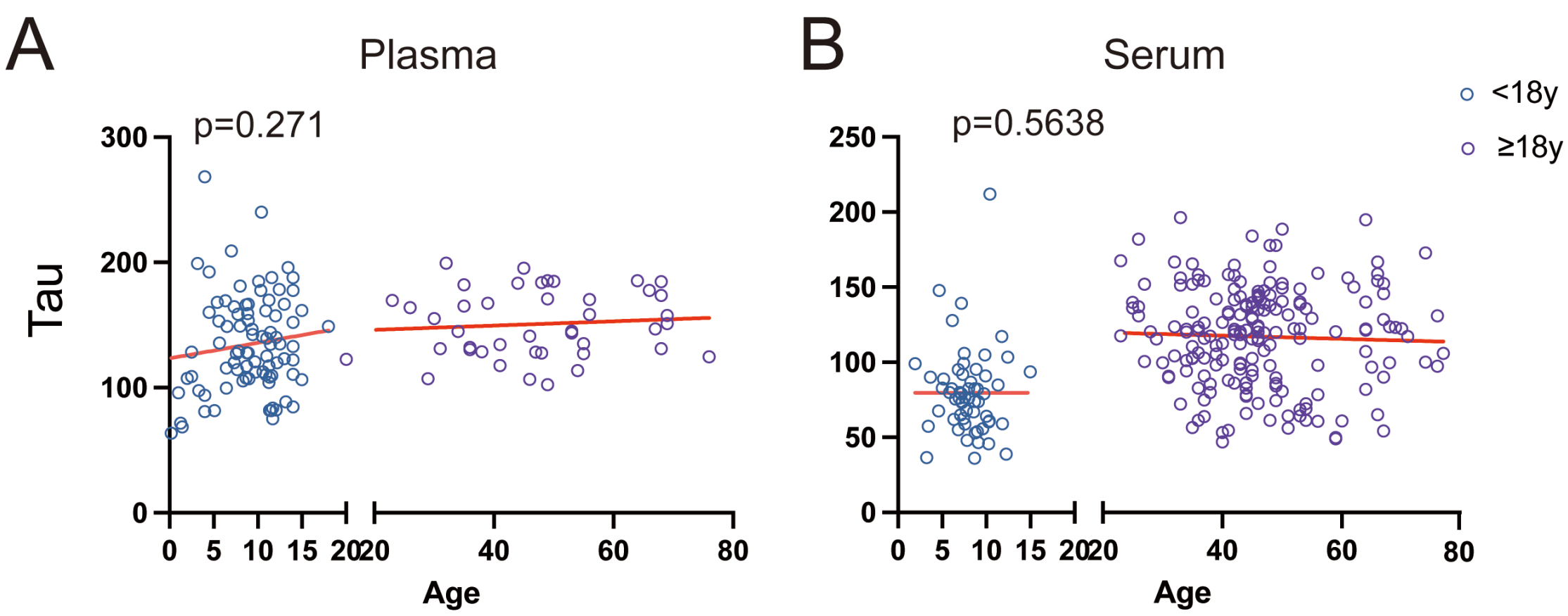


**SFigure 8.** Correlation between serum and plasma Tau and age in children and adults. (A) The correlation of plasma Tau with age in children (<18 years) and adults (≥18 years). (B) The correlation of serum Tau with age in children (<18 years) and adults (≥18 years). Tau, taurine.

## 1.9 Supplementary Table 1. Parameters of HPLC

| Time (min) | Phase |
|---|---|
| 0 | 3% MP B |
| 1.8 | 3% MP B |
| 2.5 | 30% MP B |
| 5 | 35% MP B |
| 7.5 | 85% MP B |
| 8.6 | 3% MP B |
| 9.8 | 3% MP B |

**1.10 Supplementary Table 2.** Mass spectrometry parameters of 29 amino acids

| Type | Amino | Abbr | Precursor Ion(m/z) | Product Ion(m/z) | Retention time (min) |
|---|---|---|---|---|---|
| EAAs | Phenylalanine | Phe | 337.2 | 171 | 8.2 |
| | Tryptophan | Trp | 376.2 | 171 | 8.5 |
| | Valine | Val | 290.2 | 171 | 6.7 |
| | Threonine | Thr | 290.2 | 171 | 4.6 |
| | Methionine | Met | 321.2 | 171 | 6.6 |
| | Lysine | Lys | 245 | 172 | 6.1 |
| | Leucine | Leu | 303.2 | 171 | 8.0 |
| | Isoleucine | lle | 303.2 | 171 | 7.8 |
| | Histidine (Children Specific) | His | 163.6 | 171 | 2.1 |
| Conditionally essential AA | Tyrosine | Tyr | 353.2 | 171 | 6.6 |
| NEAAs | Alanine | Ala | 261.1 | 171 | 4.9 |
| | Glutamic Acid | Glu | 319.2 | 171 | 4.4 |
| | Arginine | Arg | 173 | 171 | 3.5 |
| | Glycine | Gly | 247.2 | 171 | 3.9 |
| | Serine | Ser | 277.2 | 171 | 3.7 |
| | Proline | Pro | 288.2 | 171 | 5.3 |
| | Aspartic Acid | Asp | 304.2 | 171 | 4.3 |
| | Asparagine | Asn | 303.2 | 171 | 3.1 |
| | Glutamine | Gln | 318.2 | 171 | 3.8 |
| Other AA | Ornithine | Orn | 238 | 171 | 5.7 |
| | Taurine | Tau | 297.2 | 171 | 3.7 |
| | Hydroxyproline | Hyp | 302.2 | 171 | 2.3 |
| | Citrulline | Cit | 346.2 | 171 | 4.4 |
| | Sarcosine | Sar | 260.2 | 171 | 4.2 |
| | β-Aminoisobutyric acid | bAib | 274.2 | 171 | 5.3 |
| | α-Aminobutyric acid | Abu | 275.2 | 171 | 5.8 |
| | β-Alanine | bAla | 260.2 | 171 | 4.6 |
| | α-Aminoadipic acid | Aad | 332.2 | 171 | 5.3 |
| | γ-Aminobutyric acid | GABA | 274.2 | 171 | 5.0 |

**1.11 Supplementary Table 3.** Standard curves and quality control concentrations (µmol/L) of 29 amino acids.

| No. | Amino | S1 | S2 | S3 | S4 | S5 | S6 | QCL | QCH |
|---|---|---|---|---|---|---|---|---|---|
| 1 | Ala | 19.20 | 38.40 | 96.00 | 192.00 | 480.00 | 960.00 | 94.90 | 471.00 |
| 2 | Asp | 2.09 | 4.18 | 10.50 | 20.90 | 52.30 | 105.00 | 10.00 | 49.60 |
| 3 | Glu | 10.50 | 21.00 | 52.50 | 105.00 | 263.00 | 525.00 | 52.10 | 262.00 |
| 4 | Phe | 5.15 | 10.30 | 25.80 | 51.50 | 129.00 | 258.00 | 25.60 | 125.00 |
| 5 | Tyr | 5.10 | 10.20 | 25.50 | 51.00 | 128.00 | 255.00 | 25.60 | 122.00 |
| 6 | Trp | 4.00 | 8.00 | 20.00 | 40.00 | 100.00 | 200.00 | 19.70 | 97.20 |
| 7 | Val | 21.20 | 42.40 | 106.00 | 212.00 | 530.00 | 1060.00 | 106.00 | 500.00 |
| 8 | Arg | 4.50 | 9.00 | 22.50 | 45.00 | 113.00 | 225.00 | 22.10 | 111.00 |
| 9 | Gly | 20.40 | 40.80 | 102.00 | 204.00 | 510.00 | 1020.00 | 101.00 | 498.00 |
| 10 | Orn | 8.00 | 16.00 | 40.00 | 80.00 | 200.00 | 400.00 | 38.80 | 194.00 |
| 11 | His | 5.10 | 10.20 | 25.50 | 51.00 | 128.00 | 255.00 | 24.90 | 124.00 |
| 12 | Ser | 20.60 | 41.20 | 103.00 | 206.00 | 515.00 | 1030.00 | 102.00 | 483.00 |
| 13 | Thr | 20.00 | 40.00 | 100.00 | 200.00 | 500.00 | 1000.00 | 102.00 | 492.00 |
| 14 | Pro | 20.20 | 40.40 | 101.00 | 202.00 | 505.00 | 1010.00 | 102.00 | 472.00 |
| 15 | Met | 3.00 | 6.00 | 15.00 | 30.00 | 75.00 | 150.00 | 15.90 | 73.20 |
| 16 | Lys | 19.20 | 38.40 | 96.00 | 192.00 | 480.00 | 960.00 | 92.60 | 465.00 |
| 17 | Leu | 10.00 | 20.00 | 50.00 | 100.00 | 250.00 | 500.00 | 49.80 | 242.00 |
| 18 | Ile | 5.82 | 11.60 | 29.10 | 58.20 | 146.00 | 291.00 | 28.40 | 143.00 |
| 19 | Tau | 17.00 | 34.10 | 85.20 | 170.00 | 426.00 | 852.00 | 83.30 | 419.00 |
| 20 | Asn | 2.00 | 4.00 | 10.00 | 20.00 | 50.00 | 100.00 | 10.20 | 48.60 |
| 21 | Hyp | 2.00 | 4.00 | 10.00 | 20.00 | 50.00 | 100.00 | 9.47 | 46.70 |
| 22 | Gln | 26.50 | 53.00 | 133.00 | 265.00 | 663.00 | 1325.00 | 128.00 | 646.00 |
| 23 | Cit | 4.16 | 8.32 | 20.80 | 41.60 | 104.00 | 208.00 | 20.10 | 101.00 |
| 24 | Sar | 0.40 | 0.80 | 2.00 | 4.00 | 10.00 | 20.00 | 1.93 | 9.80 |
| 25 | bAib | 0.40 | 0.80 | 2.00 | 4.00 | 10.00 | 20.00 | 1.98 | 9.62 |
| 26 | Abu | 2.08 | 4.16 | 10.40 | 20.80 | 52.00 | 104.00 | 10.30 | 49.90 |
| 27 | bAla | 1.04 | 2.08 | 4.16 | 10.40 | 20.80 | 52.00 | 5.10 | 24.90 |
| 28 | Aad | 0.10 | 0.20 | 0.50 | 1.00 | 2.50 | 5.00 | 0.49 | 2.43 |
| 29 | GABA | 0.10 | 0.20 | 0.50 | 1.00 | 2.50 | 5.00 | 0.51 | 2.45 |

Abbr: Ala, alanine; Arg, arginine; Asn, asparagine; Asp, aspartate; Cit, citrulline; Gln, glutamine; Glu, glutamate; Gly, glycine; His, histidine; Hyp, hydroxyproline; Ile, isoleucine; Leu, leucine; Lys, lysine; Met, methionine; Orn, ornithine; Phe, phenylalanine; Pro, proline; Sar, sarcosine; Ser, serine; Tau, taurine; Thr, threonine; Trp, tryptophan; Tyr, tyrosine; Val, valine; bAla, β-alanine; Aad, α-aminoadipic acid; Abu, α-aminobutyric acid; bAib, β-aminoisobutyric acid; GABA, γ-aminobutyric acid.

1.12 **Supplementary Table 4.** For linearity, it was prepared using serial of double dilution of 29 AA for 6 points and 2 level of QCs.

| No. | Amino | Range | Equation | R squared |
|---|---|---|---|---|
| 1 | Ala | 19.2-960 µmol/L | Y = 111.7*X - 0.3840 | 0.9996 |
| 2 | Asp | 2.09-105 µmol/L | Y = 4.611*X + 0.2163 | 0.9997 |
| 3 | Glu | 10.5-525µmol/L | Y = 43.55*X + 4.547 | 0.9956 |
| 4 | Phe | 5.15-258 µmol/L | Y = 77.87*X + 0.9625 | 0.9982 |
| 5 | Tyr | 5.10-255 µmol/L | Y = 24.37*X + 0.6589 | 0.9988 |
| 6 | Trp | 4.00-200 µmol/L | Y = 14.86*X + 0.8342 | 0.9952 |
| 7 | Val | 21.2-1060 µmol/L | Y = 558.9*X - 4.796 | 0.9995 |
| 8 | Arg | 4.50-225 µmol/L | Y = 11.33*X - 1.754 | 0.9953 |
| 9 | Gly | 20.4-1020 µmol/L | Y = 1816*X - 17.64 | 0.9983 |
| 10 | Orn | 8.00-400 µmol/L | Y = 52.91*X - 1.225 | 0.9966 |
| 11 | His | 5.10-255 µmol/L | Y = 19.07*X + 1.475 | 0.9958 |
| 12 | Ser | 20.6-1030 µmol/L | Y = 1394*X - 0.03824 | 0.9992 |
| 13 | Thr | 20.0-1000 µmol/L | Y = 247.0*X - 1.999 | 0.9997 |
| 14 | Pro | 20.2-1010 µmol/L | Y = 376.9*X + 4.227 | 0.9961 |
| 15 | Met | 3.00-150 µmol/L | Y = 6.975*X + 0.6587 | 0.9961 |
| 16 | Lys | 19.2-960 µmol/L | Y = 102.3*X + 5.758 | 0.9971 |
| 17 | Leu | 10.0-500 µmol/L | Y = 52.95*X - 2.112 | 0.9997 |
| 18 | Ile | 5.82-291 µmol/L | Y = 446.8*X + 0.2621 | 0.9996 |
| 19 | Tau | 17.0-852 µmol/L | Y = 78.02*X - 1.511 | 0.999 |
| 20 | Asn | 2.00-100 µmol/L | Y = 10.10*X + 0.1759 | 0.9988 |
| 21 | Hyp | 2.00-100 µmol/L | Y = 9.482*X + 0.06853 | 0.9999 |
| 22 | Gln | 26.5-1325 µmol/L | Y = 1405*X + 7.891 | 0.9973 |
| 23 | Cit | 4.16 -208 µmol/L | Y = 14.86*X - 0.6572 | 0.9989 |
| 24 | Sar | 0.400-20.0 µmol/L | Y = 1.604*X + 0.1910 | 0.9918 |
| 25 | bAib | 0.400-20.0 µmol/L | Y = 1.364*X + 0.1647 | 0.9933 |
| 26 | Abu | 2.08-104 µmol/L | Y = 148.1*X - 0.3645 | 0.9994 |
| 27 | bAla | 1.04-52.0 µmol/L | Y = 4.968*X - 0.2267 | 0.9998 |
| 28 | Aad | 0.100-5.00 µmol/L | Y = 1.779*X + 0.02913 | 0.998 |
| 29 | GABA | 0.100-5.00 µmol/L | Y = 1.334*X - 0.02154 | 0.9999 |

Abbr: Ala, alanine; Arg, arginine; Asn, asparagine; Asp, aspartate; Cit, citrulline; Gln, glutamine; Glu, glutamate; Gly, glycine; His, histidine; Hyp, hydroxyproline; Ile, isoleucine; Leu, leucine; Lys, lysine; Met, methionine; Orn, ornithine; Phe, phenylalanine; Pro, proline; Sar, sarcosine; Ser, serine; Tau, taurine; Thr, threonine; Trp, tryptophan; Tyr, tyrosine; Val, valine; bAla, β-alanine; Aad, α-

aminoadipic acid; Abu, α-aminobutyric acid; bAib, β-aminoisobutyric acid; GABA, γ-aminobutyric acid.

## 1.14 Supplementary Table 5. Intra-Assay Coefficients of Variability of 29 AA.

| Analyte | Nominal µmol/L | Recovery Low | High | Mean | CV% | Bias% | SD |
|---|---|---|---|---|---|---|---|
| Ala | 94.90 | 89.70% | 106% | 94.6 | 5.77% | -0.32% | 5.46 |
| | 471.00 | 88.70% | 110% | 452 | 6.19% | -3.98% | 28 |
| Asp | 10.00 | 95.70% | 105% | 10.2 | 2.59% | 2.39% | 0.265 |
| | 49.60 | 93.00% | 109% | 50.1 | 3.91% | 1.05% | 1.96 |
| Glu | 52.10 | 89.70% | 114% | 54.4 | 4.43% | 4.50% | 2.41 |
| | 262.00 | 89.90% | 105% | 268 | 2.03% | 2.43% | 5.46 |
| Phe | 25.60 | 92.70% | 112% | 25.1 | 2.94% | -1.81% | 0.738 |
| | 125.00 | 93.70% | 110% | 119 | 1.44% | -4.78% | 1.71 |
| Tyr | 25.60 | 95.20% | 105% | 25.6 | 2.27% | 0.02% | 0.58 |
| | 122.00 | 89.90% | 105% | 120 | 2.04% | -1.79% | 2.45 |
| Trp | 19.70 | 94.40% | 106% | 19.4 | 1.50% | -1.59% | 0.291 |
| | 97.20 | 92.70% | 105% | 98.3 | 2.75% | 1.12% | 2.7 |
| Val | 106.00 | 95.90% | 110% | 104 | 2.12% | -2.13% | 2.2 |
| | 500.00 | 93.20% | 115% | 491 | 5.57% | -1.77% | 27.4 |
| Arg | 22.10 | 95.30% | 110% | 22.4 | 6.14% | 1.56% | 1.38 |
| | 111.00 | 88.60% | 105% | 113 | 3.04% | 2.25% | 3.45 |
| Gly | 101.00 | 92.30% | 109% | 96.8 | 4.08% | -4.18% | 3.95 |
| | 498.00 | 93.40% | 101% | 481 | 3.71% | -3.41% | 17.8 |
| Orn | 38.80 | 91.50% | 108% | 37.9 | 5.77% | -2.43% | 2.19 |
| | 194.00 | 86.60% | 102% | 185 | 4.69% | -4.89% | 8.65 |
| His | 24.90 | 91.60% | 102% | 24.5 | 4.06% | -1.71% | 0.994 |
| | 124.00 | 90.30% | 103% | 121 | 0.89% | -2.48% | 1.08 |
| Ser | 102.00 | 98.00% | 111% | 107 | 5.60% | 4.62% | 5.97 |
| | 483.00 | 94.80% | 108% | 482 | 4.35% | -0.21% | 21 |
| Thr | 102.00 | 94.10% | 110% | 102 | 6.96% | 0.30% | 7.12 |
| | 492.00 | 90.20% | 98.60% | 471 | 0.63% | -4.29% | 2.96 |
| Pro | 102.00 | 95.30% | 114% | 101 | 3.13% | -0.76% | 3.17 |
| | 472.00 | 85.90% | 113% | 491 | 7.46% | 4.09% | 36.7 |
| Met | 15.90 | 92.40% | 103% | 16 | 0.48% | 0.66% | 0.0768 |
| | 73.20 | 94.00% | 103% | 70.8 | 2.43% | -3.25% | 1.72 |
| Lys | 92.60 | 90.90% | 107% | 94.9 | 5.16% | 2.50% | 4.9 |
| | 465.00 | 92.70% | 108% | 471 | 1.75% | 1.30% | 8.26 |
| Leu | 49.80 | 93.30% | 103.90% | 48.7 | 3.23% | -2.12% | 1.57 |
| | 242.00 | 96.90% | 106% | 243 | 4.45% | 0.46% | 10.8 |
| Ile | 28.40 | 96.00% | 102% | 28.2 | 1.86% | -0.69% | 0.525 |
| | 143.00 | 91.90% | 99.80% | 137 | 1.13% | -3.92% | 1.55 |
| Tau | 83.30 | 92.90% | 110% | 84.2 | 5.74% | 1.12% | 4.83 |
| | 419.00 | 88.40% | 106% | 403 | 3.57% | -3.82% | 14.4 |
| Asn | 10.20 | 89.50% | 105% | 10.1 | 4.75% | -0.71% | 0.481 |
| | 48.60 | 89.40% | 105% | 48.4 | 6.51% | -0.42% | 3.15 |
| Hyp | 9.47 | 92.90% | 108% | 9.66 | 2.43% | 2.05% | 0.235 |
| | 46.70 | 89.90% | 105% | 46.5 | 8.40% | -0.50% | 3.9 |
| Gln | 128.00 | 94.80% | 104% | 126 | 4.89% | -1.35% | 6.18 |

| | | | | | | | |
|---|---|---|---|---|---|---|---|
| | 646.00 | 88.30% | 100% | 621 | 4.54% | -3.84% | 28.2 |
| Cit | 20.10 | 95.80% | 108% | 19.7 | 1.40% | -1.92% | 0.277 |
| | 101.00 | 90.90% | 113% | 98.5 | 3.80% | -2.51% | 3.74 |
| Sar | 1.93 | 88.40% | 107% | 1.93 | 2.92% | -0.13% | 0.0564 |
| | 9.80 | 94.20% | 107% | 9.64 | 3.03% | -1.68% | 0.292 |
| bAib | 1.98 | 91.30% | 97.50% | 1.94 | 2.16% | -1.93% | 0.0419 |
| | 9.62 | 92.70% | 107% | 9.77 | 6.02% | 1.60% | 0.588 |
| Abu | 10.30 | 96.30% | 113% | 10.7 | 6.00% | 3.51% | 0.64 |
| | 49.90 | 94.20% | 108% | 52 | 1.92% | 4.19% | 1 |
| bAla | 5.10 | 102% | 114% | 5.58 | 5.28% | 9.46% | 0.295 |
| | 24.90 | 93.30% | 106% | 25.3 | 3.86% | 1.43% | 0.976 |
| Aad | 0.49 | 98.00% | 109% | 0.5 | 5.15% | 2.03% | 0.0257 |
| | 2.43 | 85.30% | 113% | 2.47 | 9.41% | 1.82% | 0.233 |
| GABA | 0.51 | 90.60% | 102% | 2.38 | 8.14% | -2.92% | 0.194 |
| | 2.45 | 88.00% | 103% | 2.38 | 8.14% | -2.92% | 0.194 |

Abbr: Ala, alanine; Arg, arginine; Asn, asparagine; Asp, aspartate; Cit, citrulline; Gln, glutamine; Glu, glutamate; Gly, glycine; His, histidine; Hyp, hydroxyproline; Ile, isoleucine; Leu, leucine; Lys, lysine; Met, methionine; Orn, ornithine; Phe, phenylalanine; Pro, proline; Sar, sarcosine; Ser, serine; Tau, taurine; Thr, threonine; Trp, tryptophan; Tyr, tyrosine; Val, valine; bAla, β-alanine; Aad, α-aminoadipic acid; Abu, α-aminobutyric acid; bAib, β-aminoisobutyric acid; GABA, γ-aminobutyric acid.

## 1.15 **Supplementary Table 6.** Inter-Assay Coefficients of Variability of 29 AA.

| **Analyte** | **Nominal μmol/L** | **Mean** | **CV%** | **Bias%** | **SD** |
|---|---|---|---|---|---|
| Ala | 94.90 | 95.3 | 5.69% | 0.42% | 5.42 |
| | 471.00 | 459 | 6.98% | -2.55% | 32.00 |
| Asp | 10.00 | 10.3 | 3.63% | 3.00% | 0.37 |
| | 49.60 | 49.9 | 5.23% | 0.60% | 2.61 |
| Glu | 52.10 | 52.3 | 7.51% | 0.38% | 3.93 |
| | 262.00 | 255 | 5.30% | -2.67% | 13.50 |
| Phe | 25.60 | 25.8 | 7.22% | 0.78% | 1.86 |
| | 125.00 | 123 | 6.03% | -1.60% | 7.44 |
| Tyr | 25.60 | 25.5 | 3.24% | -0.39% | 0.82 |
| | 122.00 | 118 | 5.20% | -3.28% | 6.19 |
| Trp | 19.70 | 19.8 | 3.61% | 0.51% | 0.72 |
| | 97.20 | 95.5 | 4.41% | -1.75% | 4.21 |
| Val | 106.00 | 108 | 5.79% | 1.89% | 6.24 |
| | 500.00 | 520 | 5.96% | 4.00% | 31.00 |
| Arg | 22.10 | 22.5 | 4.92% | 1.81% | 1.11 |
| | 111.00 | 106 | 5.69% | -4.50% | 6.05 |
| Gly | 101.00 | 98.4 | 5.51% | -2.57% | 5.42 |
| | 498.00 | 482 | 3.03% | -3.21% | 14.60 |
| Orn | 38.80 | 38.8 | 4.43% | 0.00% | 1.72 |
| | 194.00 | 188 | 5.58% | -3.09% | 10.50 |
| His | 24.90 | 24.3 | 4.72% | -2.41% | 1.14 |
| | 124.00 | 119 | 4.72% | -4.03% | 5.63 |
| Ser | 102.00 | 105.8 | 4.32% | 3.70% | 4.57 |
| | 483.00 | 488.2 | 3.82% | 1.08% | 18.63 |
| Thr | 102.00 | 96.9 | 3.39% | -5.00% | 3.28 |
| | 492.00 | 470 | 2.72% | -4.47% | 12.80 |
| Pro | 102.00 | 104 | 6.03% | 1.96% | 6.30 |
| | 472.00 | 476 | 8.77% | 0.85% | 41.70 |
| Met | 15.90 | 16 | 3.39% | 0.63% | 0.54 |
| | 73.20 | 71.8 | 3.43% | -1.91% | 2.46 |
| Lys | 92.60 | 93.9 | 5.24% | 1.40% | 4.93 |
| | 465.00 | 459 | 4.95% | -1.29% | 22.70 |
| Leu | 49.80 | 49 | 3.38% | -1.61% | 1.65 |
| | 242.00 | 243 | 3.11% | 0.41% | 7.55 |
| Ile | 28.40 | 28.1 | 2.30% | -1.06% | 0.65 |
| | 143.00 | 138 | 2.42% | -3.50% | 3.33 |
| Tau | 83.30 | 84.3 | 5.86% | 1.20% | 4.94 |
| | 419.00 | 400 | 6.36% | -4.53% | 25.40 |
| Asn | 10.20 | 10.1 | 4.90% | -0.98% | 0.49 |
| | 48.60 | 47.6 | 5.93% | -2.06% | 2.82 |
| Hyp | 9.47 | 9.79 | 2.62% | 3.38% | 0.26 |
| | 46.70 | 48 | 1.98% | 2.78% | 0.95 |
| Gln | 128.00 | 127 | 3.47% | -0.78% | 4.41 |

| | | | | | |
|---|---|---|---|---|---|
| | 646.00 | 624 | 4.41% | -3.41% | 27.50 |
| Cit | 20.10 | 20.1 | 4.22% | 0.00% | 0.85 |
| | 101.00 | 98.7 | 6.67% | -2.28% | 6.58 |
| Sar | 1.93 | 1.89 | 6.15% | -2.07% | 0.12 |
| | 9.80 | 9.62 | 3.94% | -1.84% | 0.38 |
| bAib | 1.98 | 1.9 | 2.65% | -4.04% | 0.05 |
| | 9.62 | 9.44 | 4.47% | -1.87% | 0.42 |
| Abu | 10.30 | 10.6 | 6.09% | 2.91% | 0.65 |
| | 49.90 | 51 | 4.91% | 2.20% | 2.50 |
| bAla | 5.10 | 5.54 | 4.18% | 8.63% | 0.23 |
| | 24.90 | 24.6 | 4.04% | -1.20% | 0.99 |
| Aad | 0.49 | 0.508 | 3.52% | 3.67% | 0.02 |
| | 2.43 | 2.34 | 8.63% | -3.70% | 0.20 |
| GABA | 0.51 | 0.487 | 3.64% | -4.51% | 0.02 |
| | 2.45 | 2.38 | 5.64% | -2.86% | 0.13 |

Abbr: Ala, alanine; Arg, arginine; Asn, asparagine; Asp, aspartate; Cit, citrulline; Gln, glutamine; Glu, glutamate; Gly, glycine; His, histidine; Hyp, hydroxyproline; Ile, isoleucine; Leu, leucine; Lys, lysine; Met, methionine; Orn, ornithine; Phe, phenylalanine; Pro, proline; Sar, sarcosine; Ser, serine; Tau, taurine; Thr, threonine; Trp, tryptophan; Tyr, tyrosine; Val, valine; bAla, β-alanine; Aad, α-aminoadipic acid; Abu, α-aminobutyric acid; bAib, β-aminoisobutyric acid; GABA, γ-aminobutyric acid.

**1.16 Supplementary Table 7.** The accuracy of 29 AAs at the LLOQ.

| No. | Amino | Nominal concentration | Mean | CV% | Bias% | SD |
|---|---|---|---|---|---|---|
| 1 | Ala | 19.2 | 18.6 | 9.99% | -3.34% | 1.85 |
| 2 | Asp | 2.09 | 2.06 | 8.69% | -1.26% | 0.18 |
| 3 | Glu | 10.5 | 10.2 | 5.57% | -2.73% | 0.57 |
| 4 | Phe | 5.15 | 5.36 | 6.23% | 4.16% | 0.33 |
| 5 | Tyr | 5.1 | 4.94 | 4.91% | -3.05% | 0.24 |
| 6 | Trp | 4.0 | 4.06 | 5.77% | 1.46% | 0.23 |
| 7 | Val | 21.2 | 20.9 | 4.80% | -1.40% | 1.00 |
| 8 | Arg | 4.5 | 4.62 | 3.68% | 2.71% | 0.17 |
| 9 | Gly | 20.4 | 21.7 | 6.73% | 6.56% | 1.46 |
| 10 | Orn | 8.0 | 8.21 | 7.12% | 2.61% | 0.58 |
| 11 | His | 5.1 | 5.04 | 7.56% | -1.21% | 0.38 |
| 12 | Ser | 20.6 | 20.18 | 4.77% | -2.05% | 0.96 |
| 13 | Thr | 20.0 | 20.5 | 6.32% | 2.51% | 1.30 |
| 14 | Pro | 20.2 | 19.7 | 6.36% | -2.28% | 1.25 |
| 15 | Met | 3.0 | 2.87 | 8.14% | -4.45% | 0.23 |
| 16 | Lys | 19.2 | 19 | 5.75% | -1.07% | 1.09 |
| 17 | Leu | 10 | 10.3 | 4.21% | 3.24% | 0.44 |
| 18 | Ile | 5.82 | 5.85 | 1.42% | 0.48% | 0.08 |
| 19 | Tau | 17.0 | 16.3 | 9.40% | -4.11% | 1.53 |
| 20 | Asn | 2.0 | 1.94 | 8.60% | -3.13% | 0.17 |
| 21 | Hyp | 2.0. | 1.97 | 2.07% | -1.75% | 0.04 |
| 22 | Gln | 26.5 | 26.5 | 5.78% | -0.14% | 1.53 |
| 23 | Cit | 4.16 | 4.25 | 4.60% | 2.28% | 0.20 |
| 24 | Sar | 0.4 | 0.411 | 5.54% | 2.85% | 0.02 |
| 25 | bAib | 0.4 | 0.393 | 5.94% | -1.64% | 0.02 |
| 26 | Abu | 2.08 | 2.11 | 6.77% | 1.21% | 0.14 |
| 27 | bAla | 1.04 | 1.06 | 8.32% | 1.78% | 0.09 |
| 28 | Aad | 0.1 | 0.1 | 8.90% | 0.45% | 0.01 |
| 29 | GABA | 0.1 | 0.1 | 9.91% | 0.23% | 0.01 |

Abbr: Ala, alanine; Arg, arginine; Asn, asparagine; Asp, aspartate; Cit, citrulline; Gln, glutamine; Glu, glutamate; Gly, glycine; His, histidine; Hyp, hydroxyproline; Ile, isoleucine; Leu, leucine; Lys, lysine; Met, methionine; Orn, ornithine; Phe, phenylalanine; Pro, proline; Sar, sarcosine; Ser, serine; Tau, taurine; Thr, threonine; Trp, tryptophan; Tyr, tyrosine; Val, valine; bAla, β-alanine; Aad, α-aminoadipic acid; Abu, α-aminobutyric acid; bAib, β-aminoisobutyric acid; GABA, γ-aminobutyric acid.

1.17 **Supplementary Table 8.** Correlation of 29 AAs levels between plasma and serum samples from a subgroup of 26 participants using Pearson's r.

| Type | Amino | Pearson *r* | P (two-tailed) |
|---|---|---|---|
| EAA | Ile | 0.9833 | <0.0001 |
| | Leu | 0.9681 | <0.0001 |
| | Trp | 0.9580 | <0.0001 |
| | Val | 0.9495 | <0.0001 |
| | Met | 0.9484 | <0.0001 |
| | Lys | 0.9218 | <0.0001 |
| | Thr | 0.8994 | <0.0001 |
| | His | 0.8931 | <0.0001 |
| | Phe | 0.8170 | <0.0001 |
| Conditional essential AA | Tyr | 0.9336 | <0.0001 |
| NEAAs | Gly | 0.9353 | <0.0001 |
| | Pro | 0.9330 | <0.0001 |
| | Gln | 0.8486 | <0.0001 |
| | Ala | 0.9007 | <0.0001 |
| | Asn | 0.8610 | <0.0001 |
| | Glu | 0.9212 | <0.0001 |
| | Arg | 0.7782 | <0.0001 |
| | Ser | 0.5446 | 0.004 |
| | Asp | 0.0732 | 0.7224 |
| Other AA | bAib | 0.9913 | <0.0001 |
| | Abu | 0.9886 | <0.0001 |
| | Aad | 0.9852 | <0.0001 |
| | Hyp | 0.9812 | <0.0001 |
| | bAla | 0.9644 | <0.0001 |
| | Cit | 0.9534 | <0.0001 |
| | Orn | 0.8020 | <0.0001 |
| | GABA | 0.6606 | 0.0002 |
| | Tau | 0.4274 | 0.0294 |
| | Sar | 0.4096 | 0.0377 |

**1.18 Supplementary Table 9.** Comparison of Differences in 29 Amino Acid Levels Between Plasma and Serum.

| Amino | Plasma (Mean) | Serum (Mean) | P (two-tailed) | q value |
|---|---|---|---|---|
| Asp | 9.165 | 30.17 | <0.000001 | <0.000001 |
| Arg | 37.06 | 120.6 | <0.000001 | <0.000001 |
| Sar | 11.12 | 1.615 | <0.000001 | <0.000001 |
| Phe | 70.70 | 99.72 | <0.000001 | <0.000001 |
| GABA | 0.3862 | 0.2480 | <0.000001 | <0.000001 |
| Orn | 148.2 | 111.6 | <0.000001 | <0.000001 |
| Ser | 157.4 | 185.8 | <0.000001 | <0.000001 |
| Tau | 140.1 | 111.4 | <0.000001 | <0.000001 |
| Gly | 276.5 | 319.7 | <0.000001 | <0.000001 |
| Asn | 50.03 | 45.61 | <0.000001 | <0.000001 |
| Met | 26.45 | 29.73 | 0.000003 | 0.000003 |
| Trp | 56.25 | 60.88 | 0.000046 | 0.000050 |
| Gln | 501.0 | 534.1 | 0.000214 | 0.000217 |
| Val | 270.1 | 289.1 | 0.001139 | 0.001068 |
| bAla | 3.955 | 3.477 | 0.001522 | 0.001332 |
| Glu | 92.54 | 106.0 | 0.002006 | 0.001647 |
| Leu | 155.8 | 162.2 | 0.1079 | 0.0833 |
| Abu | 24.1 | 25.53 | 0.1143 | 0.0834 |
| His | 90.76 | 92.73 | 0.1242 | 0.0859 |
| Cit | 30.33 | 31.37 | 0.1816 | 0.1192 |
| Thr | 143.0 | 147.6 | 0.2016 | 0.1261 |
| Ile | 81.13 | 83.9 | 0.2461 | 0.1469 |
| Pro | 210.2 | 203.7 | 0.3303 | 0.1886 |
| Tyr | 75.48 | 74.1 | 0.4235 | 0.2317 |
| Ala | 447.0 | 453.4 | 0.5957 | 0.3126 |
| Aad | 0.9812 | 1.006 | 0.6206 | 0.3126 |
| Lys | 221.4 | 223.6 | 0.6428 | 0.3126 |
| Hyp | 18.61 | 19.01 | 0.7069 | 0.3315 |

Abbr: Ala, alanine; Arg, arginine; Asn, asparagine; Asp, aspartate; Cit, citrulline; Gln, glutamine; Glu, glutamate; Gly, glycine; His, histidine; Hyp, hydroxyproline; Ile, isoleucine; Leu, leucine; Lys, lysine; Met, methionine; Orn, ornithine; Phe, phenylalanine; Pro, proline; Sar, sarcosine; Ser, serine; Tau, taurine; Thr, threonine; Trp, tryptophan; Tyr, tyrosine; Val, valine; bAla, β-alanine; Aad, α-

aminoadipic acid; Abu, α-aminobutyric acid; bAib, β-aminoisobutyric acid; GABA, γ-aminobutyric acid.
